# Supplementary material for: Distance measurement between trityl radicals by pulse dressed electron paramagnetic resonance with phase modulation
Source: Magn Reson (Gott). 2020 May 15;1(1):75–87. doi: 10.5194/mr-1-75-2020 (PMC10500722; doi:10.5194/mr-1-75-2020)
Supplement: The supplement related to this article is available online at: https://doi.org/10.5194/mr-1-75-2020-supplement. [file mr-1-75-supplement.zip › mr-1-75-2020-supplement-title-page.pdf]

Supplement of Magn. Reson., 1, 75–87, 2020
https://doi.org/10.5194/mr-1-75-2020-supplement
© Author(s) 2020. This work is distributed under
the Creative Commons Attribution 4.0 License.

Open Access
MAGNETIC RESONANCE

Supplement of

Distance measurement between trityl radicals by pulse dressed electron paramagnetic resonance with phase modulation

Nino Wili et al.

Correspondence to: Nino Wili (nino.wili@phys.chem.ethz.ch)

- mr-1-75-2020-supplement-title-page.pdf
- supplement_revision_1
  - SI_partA_EPR.pdf
  - SI_partB_synthesis.pdf

The copyright of individual parts of the supplement might differ from the CC BY 4.0 License.
