# Supplementary material for: Distance measurement between trityl radicals by pulse dressed electron paramagnetic resonance with phase modulation
Source: Magn Reson (Gott). 2020 May 15;1(1):75–87. doi: 10.5194/mr-1-75-2020 (PMC10500722; doi:10.5194/mr-1-75-2020)
Supplement: The supplement related to this article is available online at: https://doi.org/10.5194/mr-1-75-2020-supplement. [file mr-1-75-supplement.zip › supplement_revision_1/SI_partB_synthesis.pdf]

Supporting Information - Part B
for
Distance measurements between trityl radicals by pulse dressed electron paramagnetic resonance with phase modulation

Nino Wili[1], Henrik Hintz[2], Agathe Vanas[1], Adelheid Godt[2], and Gunnar Jeschke[1]

[1]Department of Chemistry and Applied Biosciences, Laboratory of Physical Chemistry, ETH Zurich, Vladimir-Prelog-Weg 2, 8093 Zurich, Switzerland
[2]Faculty of Chemistry and Center for Molecular Materials (CM_2), Bielefeld University, Universitätsstrasse 25, 33615 Bielefeld, Germany

Content

Nomenclature and compound numbering
General
Synthesis of trityl-CONH-PEPBPEP-NHCO-trityl **1**
Synthesis of trityl-CONH-P(EP)$_2$B(PE)$_2$P-NHCO-trityl **2**
Influence of the trityl radical moiety on the NMR spectra
NMR data
Figures of NMR spectra

Nomenclature and compound numbering

The nomenclature used in this Supporting Information for the rulers and their building blocks is demonstrated with bis-trityl ruler **2** in Figure S1. The 1,4-phenylene, ethynylene, and butadiynylene units are abbreviated with P, E, and B (B = EE), respectively, and trityl represents the trityl moiety. This code only describes the core structural units of the compounds, but does not contain information on the side chains and other substituents. To name a concrete trityl building block, we use "trityl" for the scaffold shown in red in Figure S1 and give the other substituents in brackets.

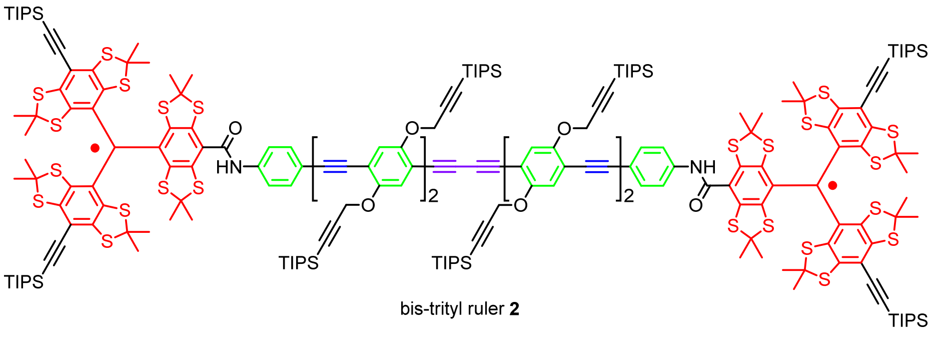

Figure S1. Illustration of the nomenclature using the example of bis-trityl ruler **2**.

Scheme 1 in the main text provides most of the compound numbers used. The additional compound number **9** belongs to the alkyne dimer shown in Figure S2.

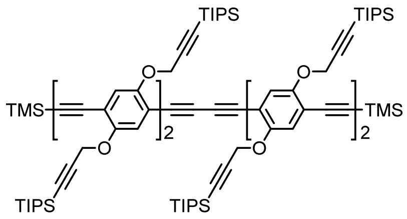

9

Figure S2. Alkyne dimer **9** isolated after Sonogashira-Hagihara coupling using TMS-$(EP)_2$E-H **3b**.

General

Unless otherwise stated, reactions were performed in dried glassware under argon. Argon was passed through anhydrous $CaCl_2$ prior to its use. Degassed solutions were prepared through applying three freeze-pump-thaw cycles. Solvents were removed at a bath temperature of ~ 40 °C and reduced pressure. The products were dried at room temperature at ~ 0.1 mbar.

Unless otherwise stated, commercially available solvents and reagents were used. Since all of these compounds which were used for syntheses had a purity of >98% their molar amounts used in the syntheses were calculated with the compound mass and were not corrected by the manufacturer specified purities. Source, purity, and batch number of the reagents can be found in Table S1. THF (HPLC grade) was dried with sodium/benzophenone prior to its use. For the preparation of aqueous solutions, deionized water was used. Solvents used for extraction and chromatography were of technical grade and were distilled prior to their use. $PdCl_2(PPh_3)_2$ was synthesized according to literature (Heck, 1985), however using 2.1 times the given amount of methanol. The syntheses of trityl[CO_2H/CCTIPS/CCTIPS] **7** (Hintz et al., 2019), TMS-$(EP)_2$E-H **3b** (Qi et al., 2016), and TMS-$(EP)_2$-NH_2 **4a** (Ritsch et al., 2019) have been reported elsewhere. Column chromatography was carried out on silica gel 60 (0.035−0.070 mm) without applying pressure. In the procedures reported below, the size of the column is given as diameter x length. The material was loaded onto the column dissolved in a small quantity of the eluent. Thin layer chromatography (TLC) was performed on silica gel 60 containing fluorescent indicator F254. The solid support for the silica gel layer was aluminum foil. The spots were detected with UV light of λ = 254 nm and 366 nm. The compositions of solvent mixtures are given in volume ratios.

NMR spectra were calibrated using the solvent signal as an internal standard [$CDCl_3$: δ (^1H) = 7.25, δ (^{13}C) = 77.0; CD_2Cl_2: δ (^1H) = 5.32, δ (^{13}C) = 53.8. Signal assignment is supported by DEPT-135, COSY, HMBC, and HMQC experiments.

ESI-MS spectra were recorded from samples dissolved in acetonitrile using an Esquire 3000 ion trap mass spectrometer (Bruker Daltonik) equipped with a standard ESI source. Accurate mass spectrometric measurement of **6b** was performed using a Q-IMS-TOF mass spectrometer Synapt G2Si (Waters, Manchester, UK) in resolution mode, interfaced

to a nano-ESI ion source. Nitrogen served as the nebulizer gas and the dry gas for nano-ESI. Nitrogen was generated by a nitrogen generator NGM 11. Helium 5.0 was used as buffer gas in the IMS entry cell, nitrogen 5.0 was used for IMS. ESI samples were introduced by static nano-ESI using in-house pulled glass emitters. Accurate MS measurements of bis-trityl rulers **1** and **2** were performed using a Bruker solariX MALDI-FTICR-MS mass spectrometer. Samples were mixed with a 10:1 mixture of *trans*-2-[3-(4-*tert*-butylphenyl)-2-methyl-2-propenylidene]malononitrile (DCTB) and dichloromethane. The monoisotopic mass of the compounds is reported.

Table S1. Source, purity, and batch number of commercially available compounds used for reactions.

compound	manufacturer	purity [%]	batch number
Chloroform, dry, over molecular sieve	Acros	99.9	1851540
Dichloromethane	VWR	99.9	15D300512
Diisopropylethylamine, dry, over molecular sieve	Merck	≥ 98	S7323294644
4-Iodoaniline	Acros	99	A0340759
2-Methylbut-3-yn-2-ol	Aldrich	98	1349654 53307311
Methanol	VWR	100.0	17G054004
Piperidine	Alfa Aesar	99	17181722
Potassium carbonate	VWR	100.6	07E020030
Thionyl chloride	Merck	≥ 99.0	S7454854804
Triphenylphosphane	Merck	≥ 99	S5329270917

Synthesis of trityl-CONH-PEPBPEP-NHCO-trityl 1 (bis-trityl ruler 1)

H-(EP)$_2$-NH$_2$ 5a. The reaction was performed under argon but without drying the glassware. To a solution of a 85:15 mixture (100 mg) of TMS-(EP)$_2$-NH$_2$ **4a** (138 µmol) and Et_2O (24 µmol) in MeOH (6 mL) and CH_2Cl_2 (6 mL) was added K_2CO_3 (29 mg, 0.21 mmol). The suspension consisting of a yellow solution and a colorless solid was stirred at room temperature for 14.5 h. Et_2O (40 mL) and H_2O (10 mL) were added. The organic phase was separated, and the aqueous phase was extracted with Et_2O (2 x 10 mL). The combined organic phases were dried over $MgSO_4 \cdot x\,H_2O$ and filtered. The solvents were removed. The components of the residual brown oil were separated by column chromatography (3 cm x 15 cm). Elution with pentane/Et_2O 1:1 gave H-(EP)$_2$-NH$_2$ **5a** (85 mg, 96%; R_f = 0.31) as a yellow oil. For ^{1}H NMR data see Table S2. For ^{13}C NMR data see Tables S3 and S4. MS (ESI): m/z = 638.5 $[M + H]^+$, 660.5 $[M + Na]^+$.

H$_2$N-PEPBPEP-NH$_2$ 6a. The reaction was performed in air. To a solution of H-(EP)$_2$-NH$_2$ **5a** (91 mg, 0.14 mmol) in dry THF (6 mL) and piperidine (706 µL, 7.13 mmol) were added $PdCl_2(PPh_3)_2$ (4.24 mg, 6.03 µmol) and CuI (4.13 mg, 21.7 µmol). The green solution was stirred at room temperature for 16 h. All volatiles were evaporated. The residual green oil was dissolved in Et_2O (30 mL). The organic phase was washed with a 0.1 M aqueous solution of Na_2H_2EDTA (3 x 10 mL). The combined aqueous phases were extracted with Et_2O (10 mL). The combined organic phases were washed with H_2O (10 mL), dried over $MgSO_4 \cdot x\,H_2O$, and filtered. The solvents were removed. The components of the residual brown solid were separated by column chromatography (2 cm x 41 cm). Eluting with Et_2O/pentane 4:1 gave a mixture of unidentified compounds (10 mg; R_f = 0.57, 0.49) as a yellow solid and H$_2$N-PEPBPEP-NH$_2$ **6a** (33 mg, 36%; R_f = 0.37) as an orange solid. For ^{1}H NMR data see Table S2. For ^{13}C NMR data see Tables S3 and S4. MS (ESI): m/z = 1273.9 $[M + H]^+$, 1295.9 $[M + Na]^+$.

Trityl-CONH-PEPBPEP-NHCO-trityl 1 (bis-trityl ruler 1). For the preparation of trityl[COCl/CCTIPS/CCTIPS] **8** the procedure of Shevelev et al. (2014) was followed with making modifications. To a solution of trityl[CO_2H/CCTIPS/CCTIPS] **7** (50 mg, 39 µmol) in dry $CHCl_3$ (5 mL) was added thionyl chloride (85.4 µL, 1.18 mmol). The red solution was stirred at 50 °C for 90 min. After cooling down to room temperature all volatiles were

evaporated from the dark red solution. The residual dark red solid was dissolved in $CHCl_3$ (4 mL). To this dark red solution cooled with an ice-water bath was added a solution of H_2N-PEPBPEP-NH_2 **6a** (20 mg, 16 µmol) in $CHCl_3$ (6 mL) and $^{i}Pr_2NEt$ (10.7 µL, 62.7 µmol). After 5 min the ice-water bath was removed and the red/brown solution was stirred for 17 h at room temperature. The brown solution was filtered through silica gel (1 cm x 2 cm, rinsing with Et_2O). The resulting solution (20 mL) was diluted with Et_2O (30 mL), washed with 2 M aqueous solution of NaOH (10 mL), 2 M aqueous solution of HCl (10 mL) and H_2O (10 mL), dried over $MgSO_4 \cdot x\ H_2O$, and filtered. The solvents were removed. The components of the residual brown solid were separated by column chromatography (3 cm x 45 cm). Elution with pentane/Et_2O 3:2 gave a mixture (12 mg; R_f = 0.73, 0.00) of unidentified compounds as a brown solid, a mixture (6 mg; R_f = 0.73, 0.58) of trityl-CONH-PEPBPEP-NHCO-trityl **1** and unidentified compounds as a green/brown solid, and trityl-CONH-PEPBPEP-NHCO-trityl **1** (bis-trityl ruler **1**; 24 mg after freeze-drying with benzene, 40%; R_f = 0.58) as a green solid. For ^{1}H NMR data see Table S2. Accurate MS (MALDI): *m/z* calcd for $[M + H]^+$, $C_{200}H_{263}N_2O_6S_{24}Si_8^+$, 3780.1787; found, 3780.1884.

Synthesis of trityl-CONH-P(EP)$_2$B(PE)$_2$P-NHCO-trityl 2 (bis-trityl ruler 2)

TMS-(EP)$_3$-NH$_2$ 4b. To a degassed solution of TMS-(EP)$_2$E-H **3b** (200 mg, 175 µmol) and 4-iodoaniline (42 mg, 0.19 mmol) in dry THF (7 mL) and piperidine (0.87 mL, 8.8 mmol) were added $PdCl_2(PPh_3)_2$ (2.51 mg, 3.58 µmol) and CuI (1.29 mg, 6.77 µmol). The orange solution was stirred at room temperature for 46 h. Then, 2-methylbut-3-yn-2-ol (9 µL, 90 µmol) was added and the suspension was stirred for 24 h. All volatiles were evaporated. The residual viscous brown liquid was filtered through silica gel (2 cm x 3 cm, rinsing with Et_2O). The solvents were removed. The components of the residual brown oil were separated by column chromatography (3 cm x 31 cm). Eluting with pentane/Et_2O 3:1 gave as a first fraction a mixture (19 mg; R_f (pentane/Et_2O 3:1) = 0.77; R_f (pentane/Et_2O 1:1) = 0.80) of alkyne dimer **9**, grease, and a small amount of unidentified compounds as yellow solid. As the second fraction a mixture (8 mg; R_f (pentane/Et_2O 3:1) = 0.43; R_f (pentane/Et_2O 1:1) = 0.74) of unidentified compounds, Et_2O, and grease was obtained as

a yellow oil. As the third fraction a mixture (16 mg; R_f (pentane/Et_2O 3:1) = 0.09; R_f (pentane/Et_2O 1:1) = 0.34) of unidentified compounds, Et_2O, and pentane was obtained as a yellow oil. The eluent was changed to pentane/Et_2O 1:1 and TMS-(EP)$_3$-NH_2 **4b** (186 mg, 86%; R_f (pentane/Et_2O 3:1) = 0.09; R_f (pentane/Et_2O 1:1) = 0.34) was obtained as a yellow solid. For ^{1}H NMR data of TMS-(EP)$_3$-NH_2 **5b** and alkyne dimer **9** see Table S2. For ^{13}C NMR data of TMS-(EP)$_3$-NH_2 **5b** see Tables S3 and S4. MS (ESI) of TMS-(EP)$_3$-NH_2 **5b**: *m*/*z* = 1230.6 $[M + H]^+$, 1252.6 $[M + Na]^+$.

H-(EP)$_3$-NH$_2$ 5b. The reaction was performed under argon but without drying the glassware. To a solution of TMS-(EP)$_3$-NH_2 **4b** (150 mg, 122 µmol) in MeOH (6 mL) and CH_2Cl_2 (6 mL) was added K_2CO_3 (25 mg, 0.18 mmol). The suspension consisting of a yellow solution and a colorless solid was stirred at room temperature for 14.5 h. Et_2O (40 mL) and H_2O (10 mL) were added. The organic phase was separated, and the aqueous phase was extracted with Et_2O (2 x 10 mL). The combined organic phases were dried over $MgSO_4 \cdot x\ H_2O$ and filtered. The solvents were removed. The components of the residual orange solid were separated by column chromatography (3 cm x 14 cm). Elution with pentane/Et_2O 1:1 gave H-(EP)$_3$-NH_2 **5b** (135 mg, 96%; R_f = 0.51) as a yellow solid. For ^{1}H NMR data see Table S2. For ^{13}C NMR data see Tables S3 and S4. MS (ESI): *m*/*z* = 1158.8 $[M + H]^+$, 1180.8 $[M + Na]^+$.

H$_2$N-P(EP)$_2$B(PE)$_2$P-NH$_2$ 6b. The reaction was performed in air. To a solution of H-(EP)$_3$-NH_2 **5b** (133 mg, 115 µmol) in dry THF (6 mL) and piperidine (568 µL, 5.74 mmol) were added $PdCl_2(PPh_3)_2$ (3.53 mg, 5.04 µmol) and CuI (1.64 mg, 8.59 µmol). The green solution was stirred at room temperature for 15.5 h. All volatiles were evaporated. The residual green oil was dissolved in Et_2O (30 mL). The organic phase was washed with a 0.1 M aqueous solution of Na_2H_2EDTA (3 x 10 mL). The combined aqueous phases were extracted with Et_2O (10 mL). The combined organic phases were washed with H_2O (10 mL), dried over $MgSO_4 \cdot x\ H_2O$, and filtered. The solvents were removed. The components of the residual orange solid were separated by column chromatography (2 cm x 46 cm). Eluting with Et_2O/pentane 4:1 gave a mixture (10 mg; R_f = 0.71, 0.43) of unidentified compounds and a small amount of H_2N-P(EP)$_2$B(PE)$_2$P-NH_2 **6b** as a yellow solid. Then, H_2N-P(EP)$_2$B(PE)$_2$P-NH_2 **6b** (87 mg, 65%; R_f = 0.43) was obtained as yellow solid. For ^{1}H NMR data see Table S2. For ^{13}C NMR data see Tables S3 and S4. Accurate

MS (ESI): *m/z* calcd for $[M + H + Na]^{2+}$, $C_{144}H_{205}N_2NaO_8Si_8^{2+}$, 1168.6868; found, 1168.6870.

Trityl-CONH-P(EP)$_2$B(PE)$_2$P-NHCO-trityl 2 (bis-trityl ruler 2). For the preparation of trityl[CO_2Cl/CCTIPS/CCTIPS] **8** the procedure of Shevelev et al. (2014) was followed with making modifications. To a solution of trityl[CO_2H/CCTIPS/CCTIPS] **7** (28 mg, 22 µmol) in dry $CHCl_3$ (5 mL) was added thionyl chloride (47.0 µL, 684 µmol). The red solution was stirred at 50 °C for 90 min. After cooling down to room temperature all volatiles were evaporated. The residual red/brown solid was dissolved in $CHCl_3$ (5 mL). To this dark red solution cooled with an ice-water bath was added a solution of H_2N-P(EP)$_2$B(PE)$_2$P-NH_2 **6b** (20 mg, 8.6 µmol) in $CHCl_3$ (5 mL) and $^iPr_2NEt$ (5.9 µL, 34.7 µmol). After 10 min the ice-water bath was removed, and the red solution was stirred for 19 h at room temperature. The brown solution containing a small amount of colorless solid was diluted with Et_2O (30 mL), washed with a 2 M aqueous solution of HCl (10 mL) and H_2O (10 mL), dried over $MgSO_4 \cdot x\ H_2O$, and filtered. The filtrate was filtered through silica gel (2 cm x 3 cm, rinsing with Et_2O). The solvents were removed. The components of the residual green solid were separated by column chromatography (3 cm x 45 cm). Elution with pentane/Et_2O 7:3 gave a mixture (5 mg; R_f = 0.88) of unidentified compounds as a brown solid, trityl-CONH-P(EP)$_2$B(PE)$_2$P-NHCO-trityl **2** (bis-trityl ruler **2**; 24 mg after freeze-drying with benzene, 58%; R_f = 0.66) as a green solid, and a 63:29:8 mixture (4 mg, R_f = 0.66) of pentane, Et_2O, and trityl-CONH-P(EP)$_2$B(PE)$_2$P-NHCO-trityl **2** (8% yield) as a green/brown solid. For ^1H NMR data see Table S2. Accurate MS (MALDI): *m/z* calcd for $[M + {}^iPrOH + H]^+$, $C_{267}H_{367}N_2O_{11}S_{24}Si_{12}^+$, 4880.8748; found, 4880.8712.

Influence of the trityl radical moiety on the NMR spectra

Because it is paramagnetic, the trityl radical moiety takes a distance dependent influence on the NMR signals of the compounds. In Figure S3 a cutout of the bis-trityl rulers **1** and **2** is shown. The signals of the hydrogen atoms in the area marked with red are not found in the ^{1}H NMR spectra. The signals of hydrogen atoms in the green highlighted area are observed and are sometimes broadened.

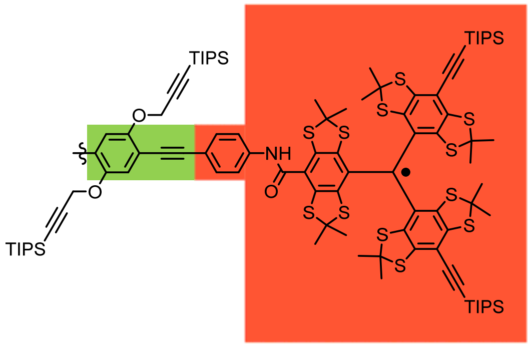

Figure S3. Cutout of the bis-trityl rulers **1** and **2**. Hydrogen atoms, whose NMR signals are affected by the unpaired electron spin, are marked with colors.

NMR data

Table S2. ^{1}H NMR (500 MHz) data.

**compound**, solvent	H_{benz} *ortho* to NH_2	H_{benz} *ortho* to OCH_2	H_{benz} *meta* to NH_2	$C_{benz}OCH_2$	NH_2	C≡CH	$SiCHMe_2$	$SiMe_3$
**1** CD_2Cl_2	---	7.31 (s, 2H), 7.21 (br s, 2H)	---	4.85, 4.84, both s with large overlap, together 8H	---	---	1.08 (s, 42H), 1.06 (s, 42H)	---
**2** CD_2Cl_2	---	7.32 (s, 2H), 7.29 (br s, 2H), 7.25 (s) overlapping with 7.23 (br s) together 4H	---	4.87, 4.86, 4.84, all s with large overlap, together 16H	---	---	1.07, 1.06, both s with large overlap, together 168H	---
**4b** CD_2Cl_2	7.31 (AA'XX' spin system, 2H)	7.29 (s, 1H), 7.26 (s, 1H), 7.19 (s, 1H), 7.17 (s, 1H)	6.65 (AA'XX' spin system, 2H)	4.85 (s, 2H), 4.84 (s, 2H), 4.81 (s, 2H), 4.78 (s, 2H)	3.95 (s, 2H)	---	1.063, 1.062, 1.058, all s with large overlap, together 84H	0.26 (s, 9H)
**5a** CD_2Cl_2	7.31 (AA'XX' spin system, 2H)	7.25 (s, 1H), 7.21 (s, 1H)	6.64 (AA'XX' spin system, 2H)	4.80 (s, 2H), 4.79 (s, 2H)	3.95, 3.93, both s with large overlap, together 1.7H[#]	3.38 (s, 1H)	1.066 (s, 21H), 1.065 (s, 21H)	---
**5b** CD_2Cl_2	7.32 (AA'XX' spin system, 2H)	7.30 (s, 1H), 7.29 (s, 1H), 7.23 (s, 1H), 7.20 (s, 1H)	---	4.854 (s, 2H), 4.848 (s, 2H), 4.82 (s, 2H), 4.80 (s, 2H)	3.95, 3.93, both s with overlap, together 1.7H[#]	3.40 (s, 1H)	1.07, 1.061, 1.059, 1.056, all s with large overlap, together 84H	---
**6a** CD_2Cl_2	7.31 (AA'XX' spin system, 4H)	7.27 (s, 2H), 7.22 (s, 2H)	6.64 (AA'XX' spin system, 4H)	4.82 (s, 4H), 4.80 (s, 4H)	3.95, 3.93, both s with large overlap, together 1.9H[##]	---	1.07 (s, 42H), 1.05 (s, 42H)	---
**6b** CD_2Cl_2	7.32 (AA'XX' spin system, 4H)	7.31 (s, 2H), 7.30 (s, 2H), 7.26 (s, 2H), 7.20 (s, 2H)	---	4.861, 4.858, both s with large overlap, together 8H, 4.84 (s, 4H), 4.82 (s, 4H)	3.95 (s, 4H)	---	1.073 (s, 42H), 1.069 (s, 42H), 1.061 (s, 42H), 1.055 (s, 42H)	---
**9** $CDCl_3$	---	7.27 (s, 2H), 7.25 (s, 2H), 7.22 (s, 2H), 7.15 (s, 2H)	---	4.82 (s, 4H), 4.80 (s, 4H), 4.79 (s, 4H), 4.75 (s, 4H)	---	---	1.040, 1.036, both s with large overlap, together 126H; 1.02 (s, 42H)	0.25 (s, 18H)

benz = benzene; [#]A singlet with an integral corresponding to 2H is expected. Partial exchange with deuterium led to two singlets assigned to NH_2 and NDH. [##]A singlet with an integral corresponding to 4H is expected. Partial exchange with deuterium led to two singlets assigned to NH_2 and NDH.

Table S3. ^{13}C NMR (125 MHz) data: signals with a chemical shift above 70 ppm.

**compound**, solvent	$C_{benz}O$	$C_{benz}NH_2$	$C_{benz}H$ *meta* to $C_{benz}NH_2$	$C_{benz}H$ *meta* to $C_{benz}NH$*	$C_{benz}H$ *ortho* to $C_{benz}NH$*	C_{benz} *para* to $C_{benz}NH$*	$C_{benz}H$ *ortho* to $C_{benz}O$	$C_{benz}C≡C$ (*benz* = substituted alkoxybenzene)	$C_{benz}H$ *ortho* to $C_{benz}NH_2$	C_{benz} *para* to $C_{benz}NH_2$	$CH_2C≡C$	$C_{benz}C≡C$ *para* to $C_{benz}NH$*	$CH_2C≡C$	$C_{benz}C≡CC_{benz}$ (*benz* = substituted alkoxybenzene)	$C_{benz}C≡C$ *para* to $C_{benz}NH_2$	C≡C-C≡C	TMSC≡C	C≡CH
**4b** CD_2Cl_2	153.1, 152.9, 152.6, 152.5	147.7	133.3	---	---	---	120.3, 119.4, 119.1, 119.0	115.8, 115.0, 114.5, 113.6,	114.8	112.4	102.2, [102.15, 102.14], 101.9	---	90.6, 90.5, 90.2, 90.1	96.8, 91.8, 91.1, 83.7		---	[100.87, 100.86]	---
**5a** CD_2Cl_2	153.5, 152.3	147.8	133.3	---	---	---	120.0, 118.7	116.2, 112.3	114.8	112.3	102.1, 102.0	96.7, 83.4	90.5, 90.2	---	---	---	---	82.7, 79.9
**5b** CD_2Cl_2	153.5, 153.0, 152.6, 152.5	147.7	133.3	---	---	---	120.6, 119.4, 119.1, 118.8	115.9, 115.5, 113.6, 113.3	114.9	112.4	102.3, 102.2, 102.1, 101.9	---	90.6, 90.5*^{2}, 90.1	96.8, 91.8, 90.9, 83.7		---	---	83.1, 79.8
**6a** CD_2Cl_2	154.4, 152.2	147.8	133.4	---	---	---	119.8, 118.6	116.8, 111.9	114.8	112.3	102.0, 101.9	---	90.7, 90.4	---	97.5, 83.7, 79.6, 79.3		---	---
**6b** CD_2Cl_2	154.4, 153.0, 152.53, 152.47	147.7	133.3	---	---	---	120.5, 119.4, 119.1, 118.7	116.1, 115.9, 113.6, 112.9	114.9	112.4	102.2, 102.1, 102.0, 101.8	---	90.8, 90.7, 90.5, 90.1	96.9, 92.5, 91.1, 83.7, 79.7, 79.6			---	---

benz = benzene. Signals reported in square brackets overlap. *^{n} means the signal has *n*-fold intensity compared to the other signals in the series.

Table S4. ^{13}C NMR (125 MHz) data: signals with a chemical shift below 70 ppm.

**compound**, solvent	$CH_2C≡C$	$CHMe_2$	$CHMe_2$	$SiMe_3$
**4b** CD_2Cl_2	58.6, 58.5	18.7	[11.51, 11.49]	0.0
**5a** CD_2Cl_2	58.6, 58.3	[18.68, 18.67]	11.5	---
**5b** CD_2Cl_2	58.7, 58.6*^{2}, 58.4	[18.72, 18.70, 18.69]	[11.52, 11.50, 11.49]	---
**6a** CD_2Cl_2	58.6, 58.3	18.7	[11.52, 11.49]	---
**6b** CD_2Cl_2	58.7, [58.59, 58.58], 58.4	18.70 with shoulder at 18.71	[11.52*^{3}, 11.49]	---

Signals reported in square brackets overlap. *^{n} means the signal has *n*-fold intensity compared to the other signals in the series.

References

Heck, R. F.: Palladium reagents in organic syntheses, Best synthetic methods, Academic Press, London, 1985.

Hintz, H., Vanas, A., Klose, D., Jeschke, G., and Godt, A.: Trityl Radicals with a Combination of the Orthogonal Functional Groups Ethyne and Carboxyl, J. Org. Chem., 84, 3304–3320, https://doi.org/10.1021/acs.joc.8b03234, 2019.

Qi, M., Hülsmann, M., and Godt, A.: Spacers for Geometrically Well-Defined Water-Soluble Molecular Rulers and Their Application, J. Org. Chem., 81, 2549–2571, https://doi.org/10.1021/acs.joc.6b00125, 2016.

Ritsch, I., Hintz, H., Jeschke, G., Godt, A., and Yulikov, M.: Improving the accuracy of Cu(II)-nitroxide RIDME in the presence of orientation correlation in water-soluble Cu(II)-nitroxide rulers, Phys. Chem. Chem. Phys., 21, 9810–9830, https://doi.org/10.1039/C8CP06573J, 2019.

Shevelev, G. Y., Krumkacheva, O. A., Lomzov, A. A., Kuzhelev, A. A., Rogozhnikova, O. Y., Trukhin, D. V., Troitskaya, T. I., Tormyshev, V. M., Fedin, M. V., Pyshnyi, D. V., and Bagryanskaya, E. G.: Physiological-Temperature Distance Measurement in Nucleic Acid using Triarylmethyl-Based Spin Labels and Pulsed Dipolar EPR Spectroscopy, J. Am. Chem. Soc., 136, 9874–9877, https://doi.org/10.1021/ja505122n, 2014.

Figures of NMR spectra

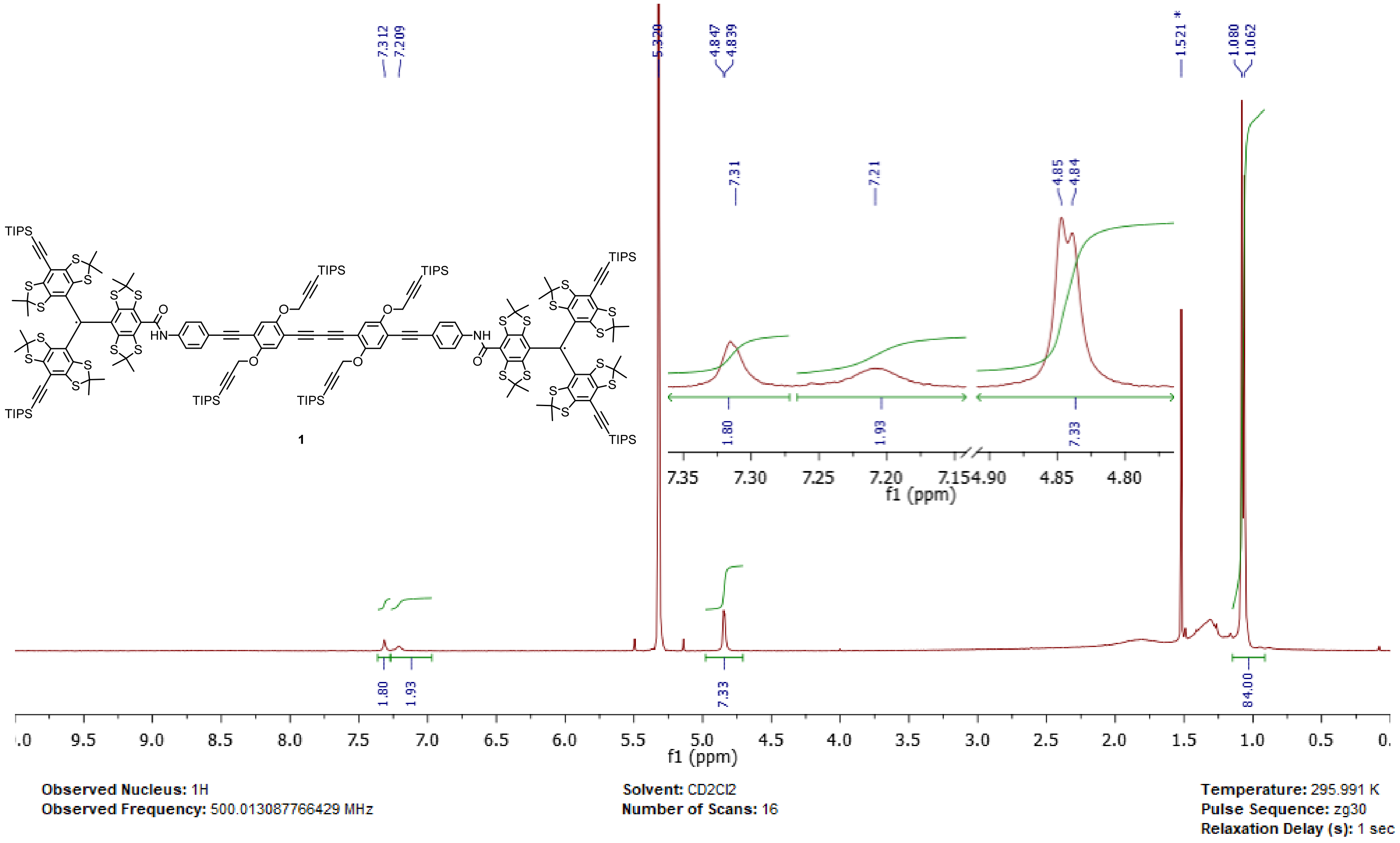

Figure S4. ^{1}H NMR spectrum of trityl-PEPBPEP-trityl **1**. *H_2O.

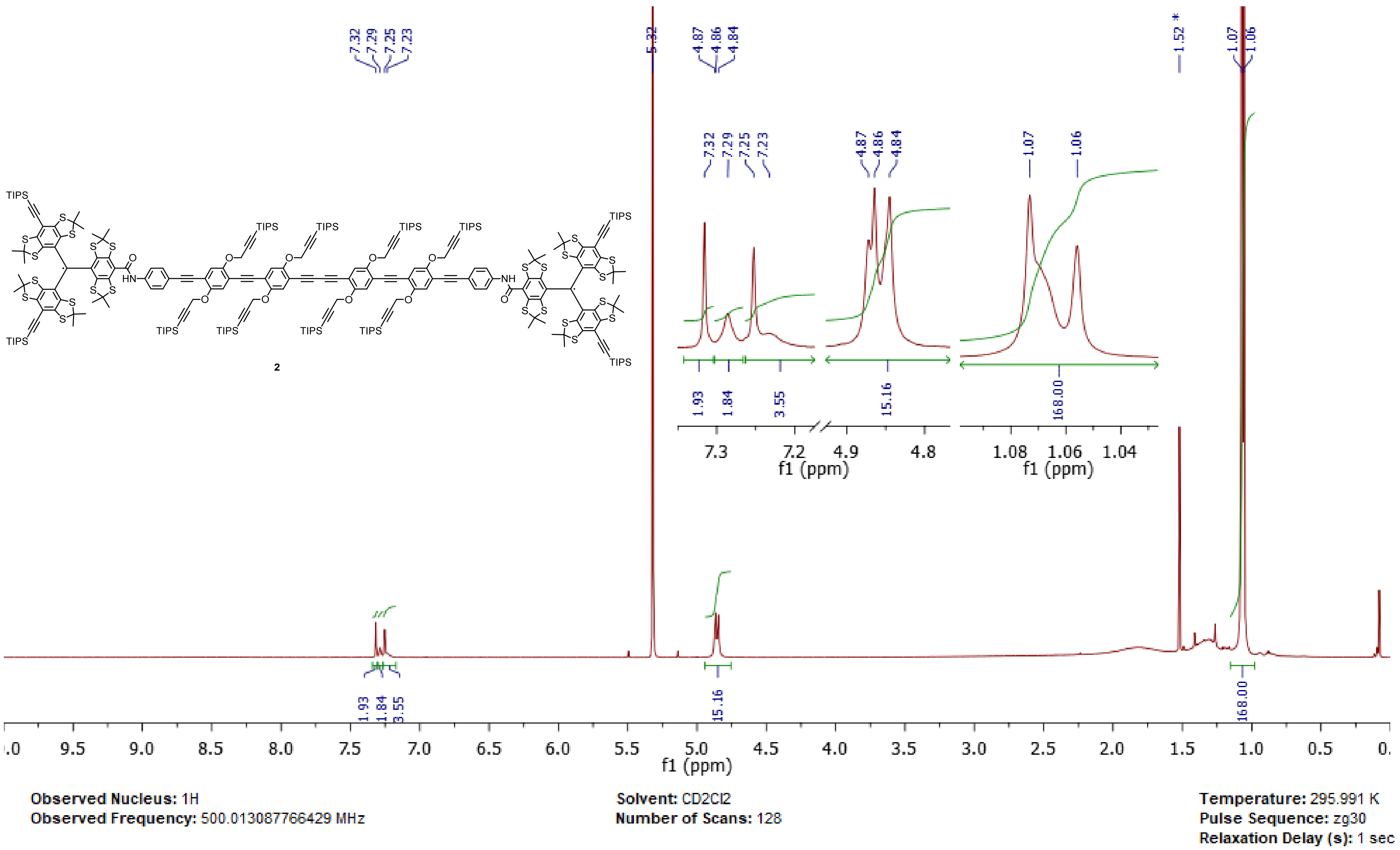

Figure S5. ^{1}H NMR spectrum of trityl-P(EP)$_2$BP(EP)$_2$-trityl **2**. *H_2O.

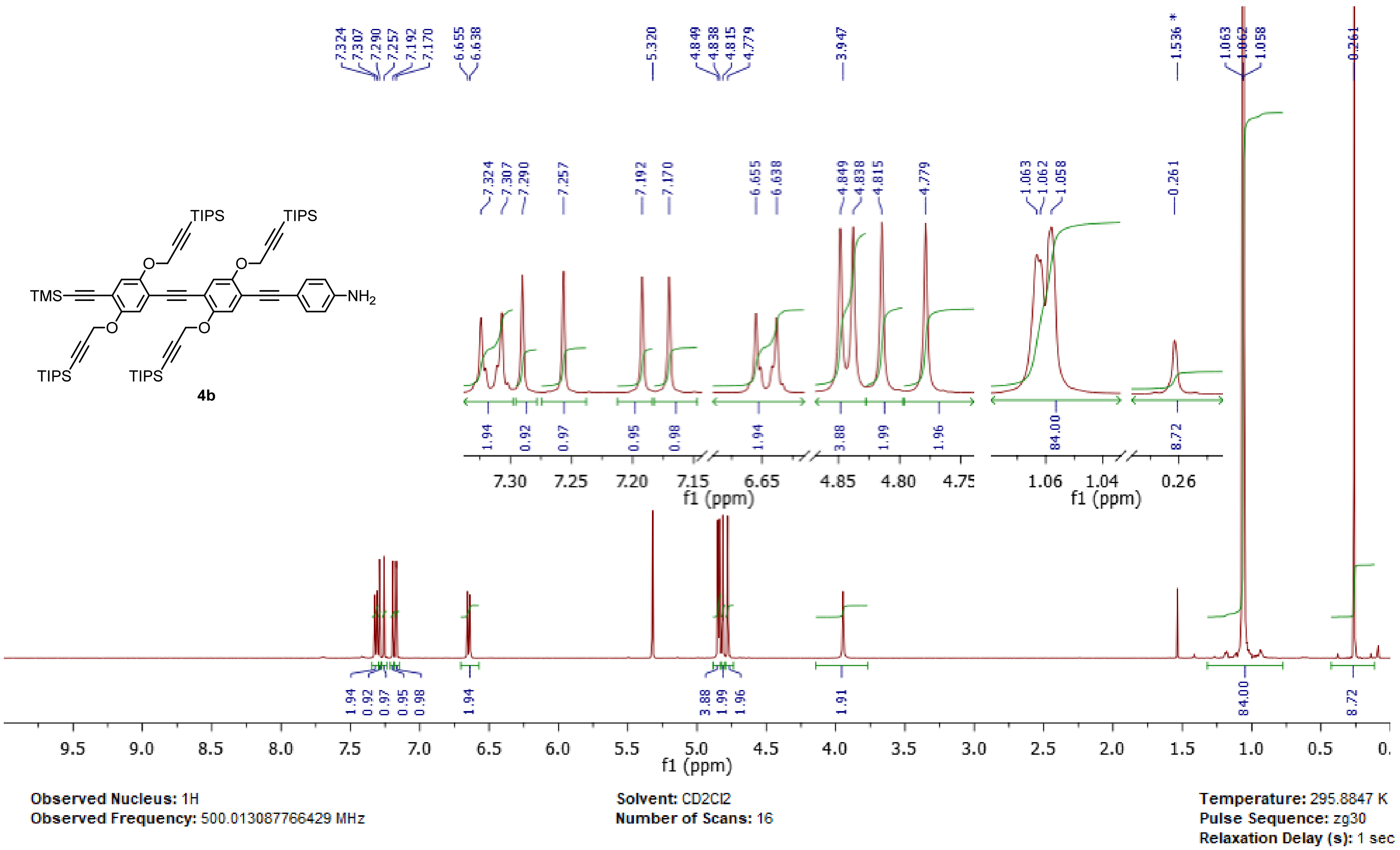

Figure S6. ^{1}H NMR spectrum of TMS-(EP)$_3$-NH_2 **4b**. *H_2O.

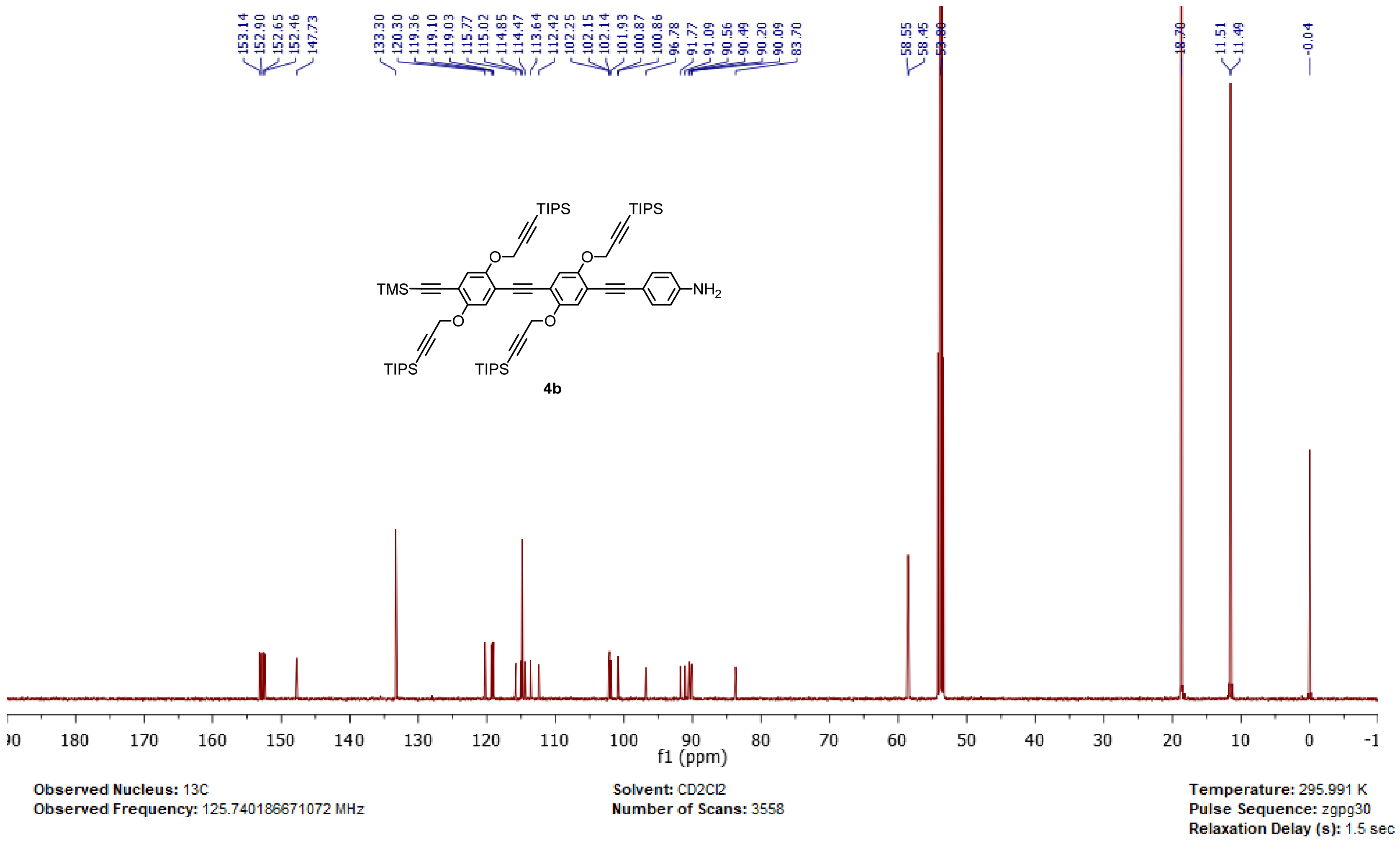

Figure S7. ^{13}C NMR spectrum of TMS-(EP)$_3$-NH_2 **4b**.

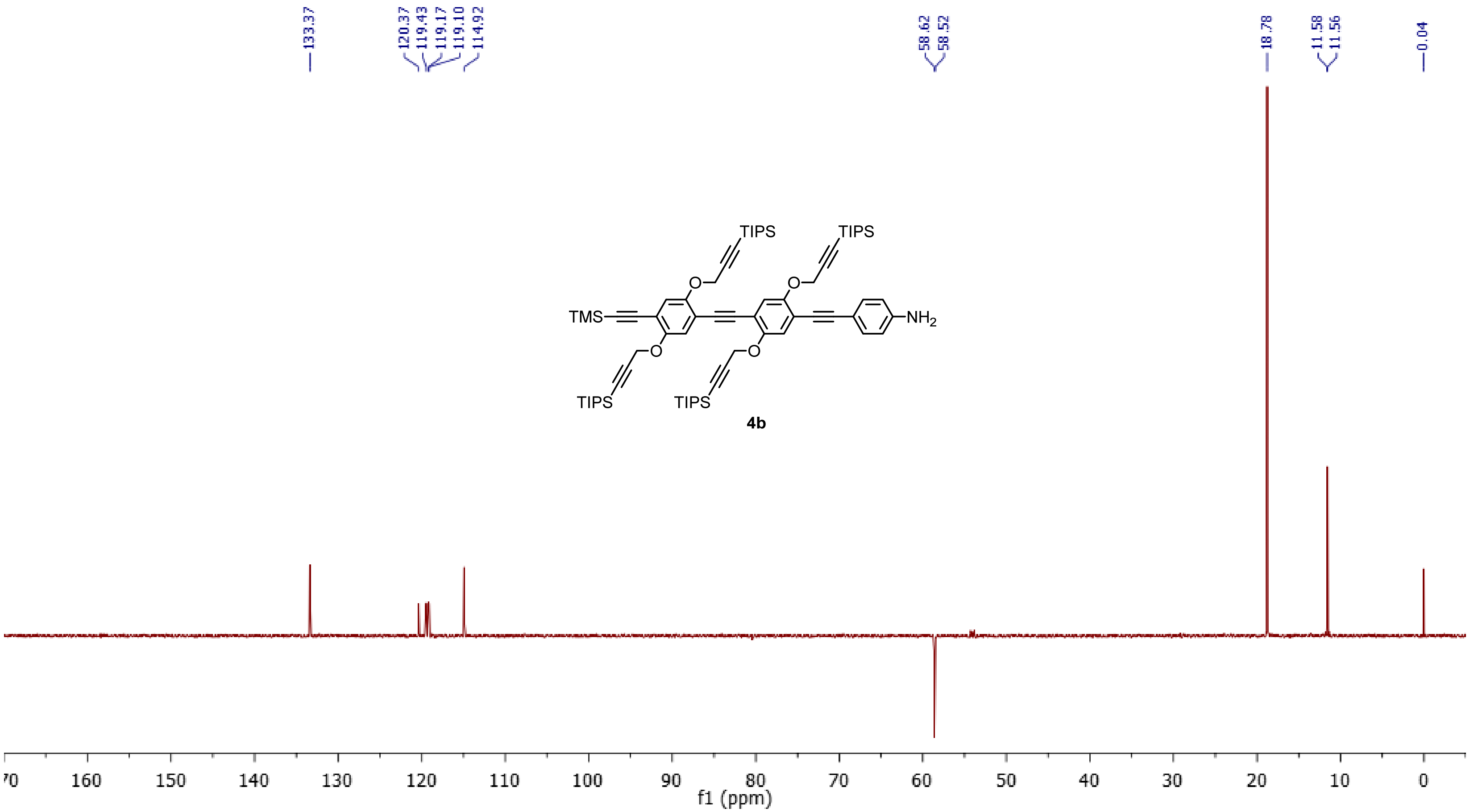

Observed Nucleus: 13C
Observed Frequency: 125.73767221312 MHz

Solvent: CD2Cl2
Number of Scans: 240

Temperature: 295.991 K
Pulse Sequence: dept135
Relaxation Delay (s): 1.5 sec

Figure S8. DEPT-135 NMR spectrum of TMS-(EP)$_3$-NH$_2$ **4b**.

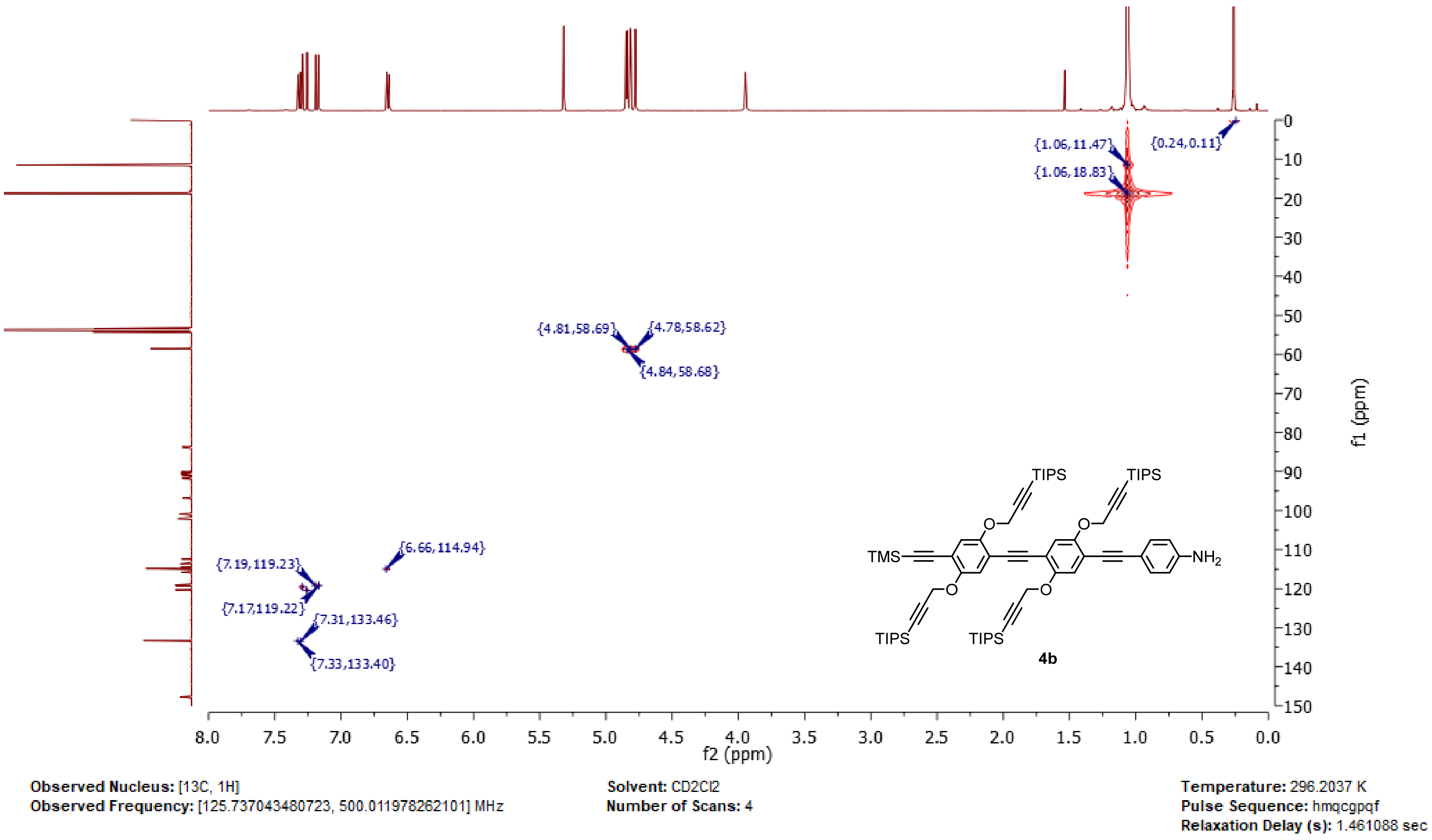

Figure S9. HMQC NMR spectrum of TMS-(EP)$_3$-NH$_2$ **4b**.

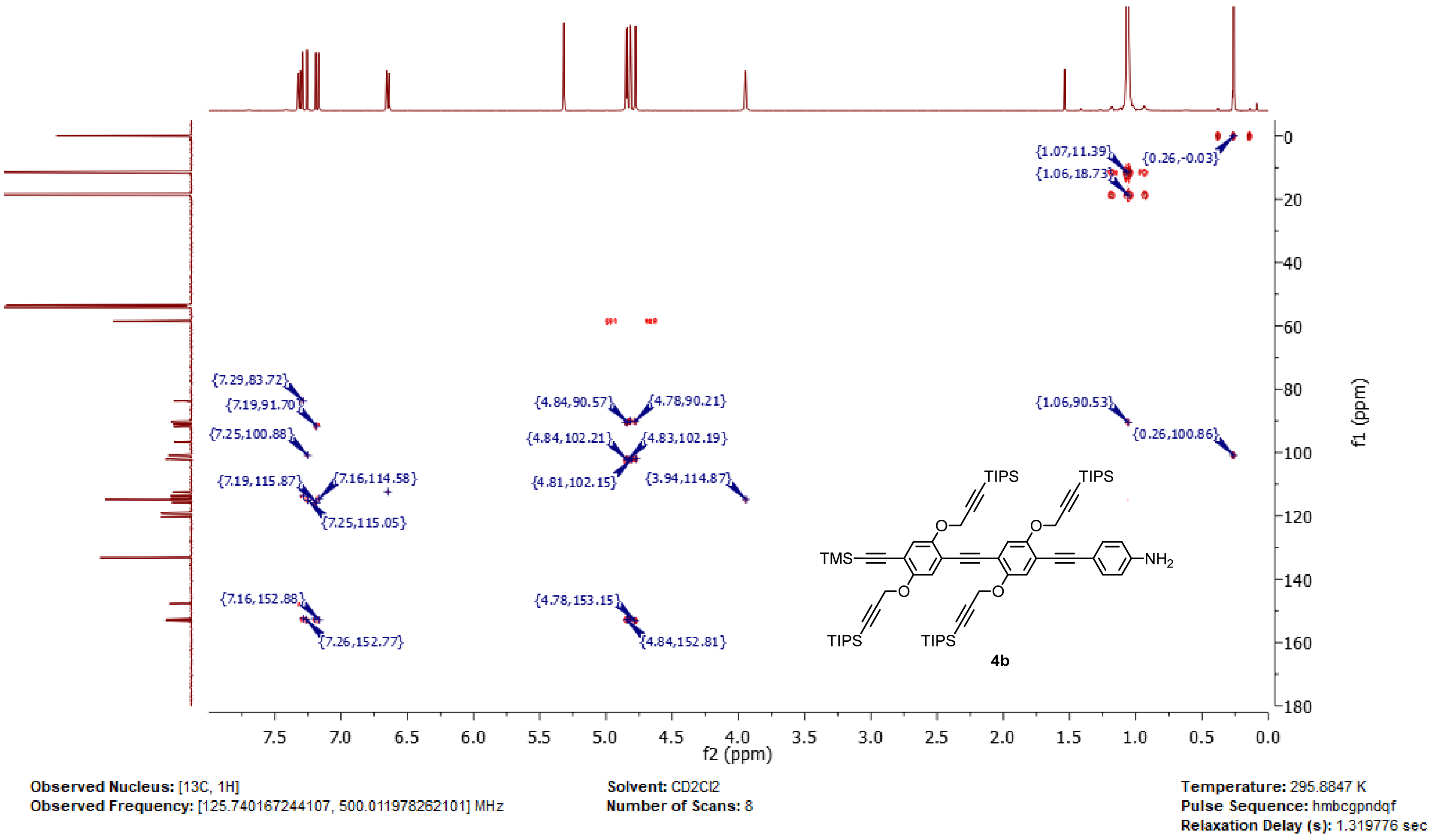

Figure S10. HMBC NMR spectrum of TMS-(EP)$_3$-NH$_2$ **4b**.

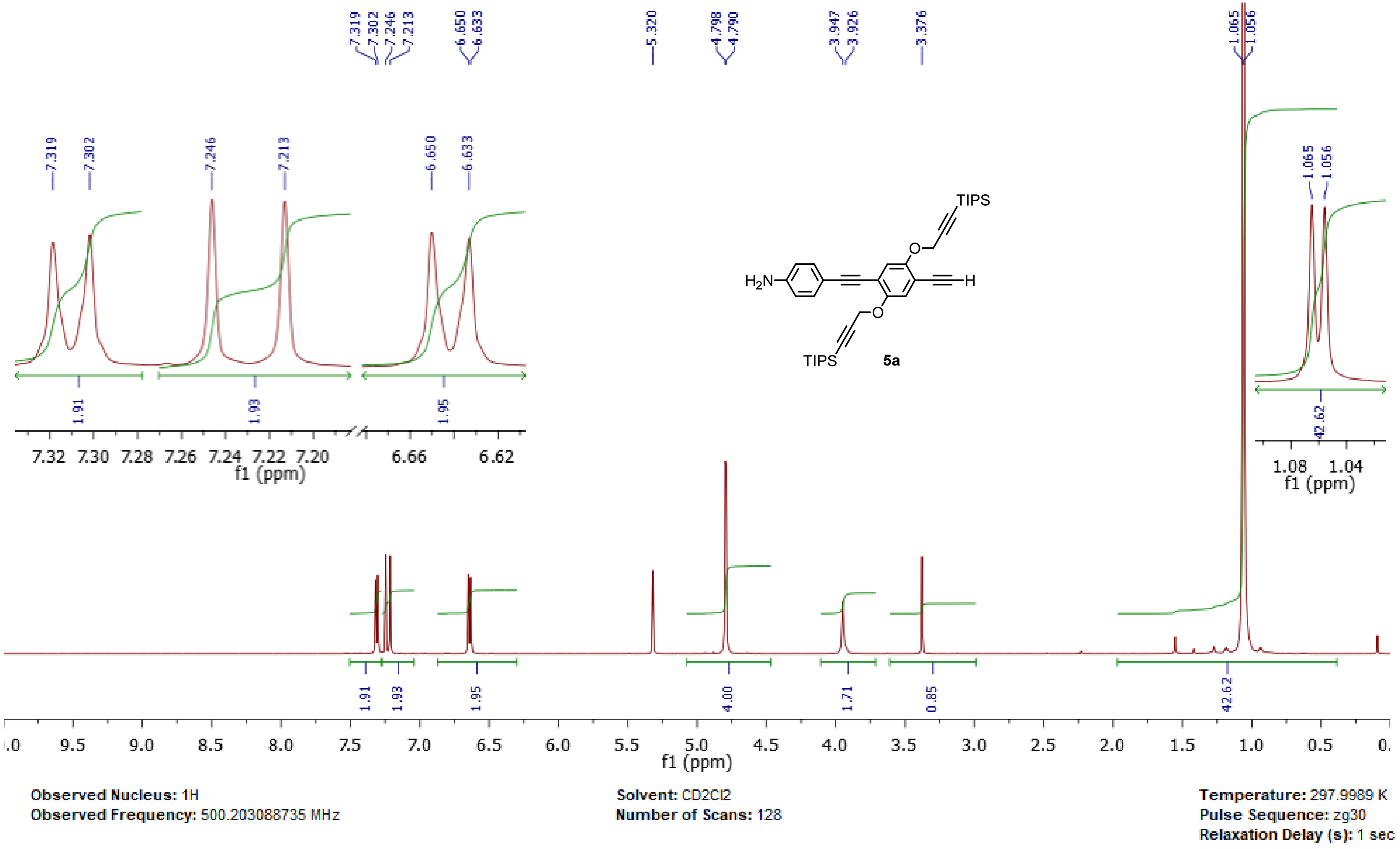

Figure S11. ^{1}H NMR spectrum of H-(EP)$_2$-NH$_2$ **5a**.

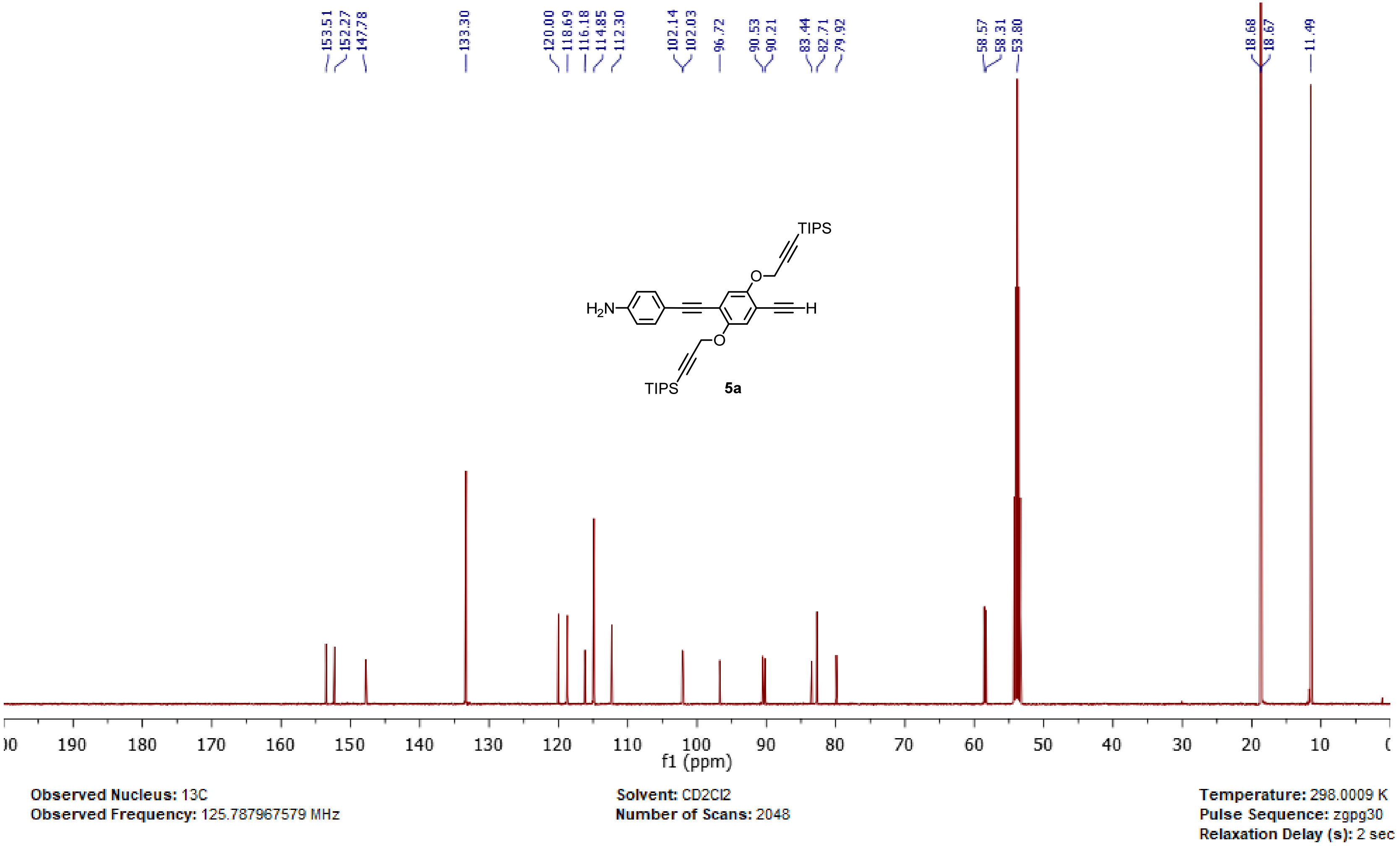

Figure S12. ^{13}C NMR spectrum of H-(EP)$_2$-NH_2 **5a**.

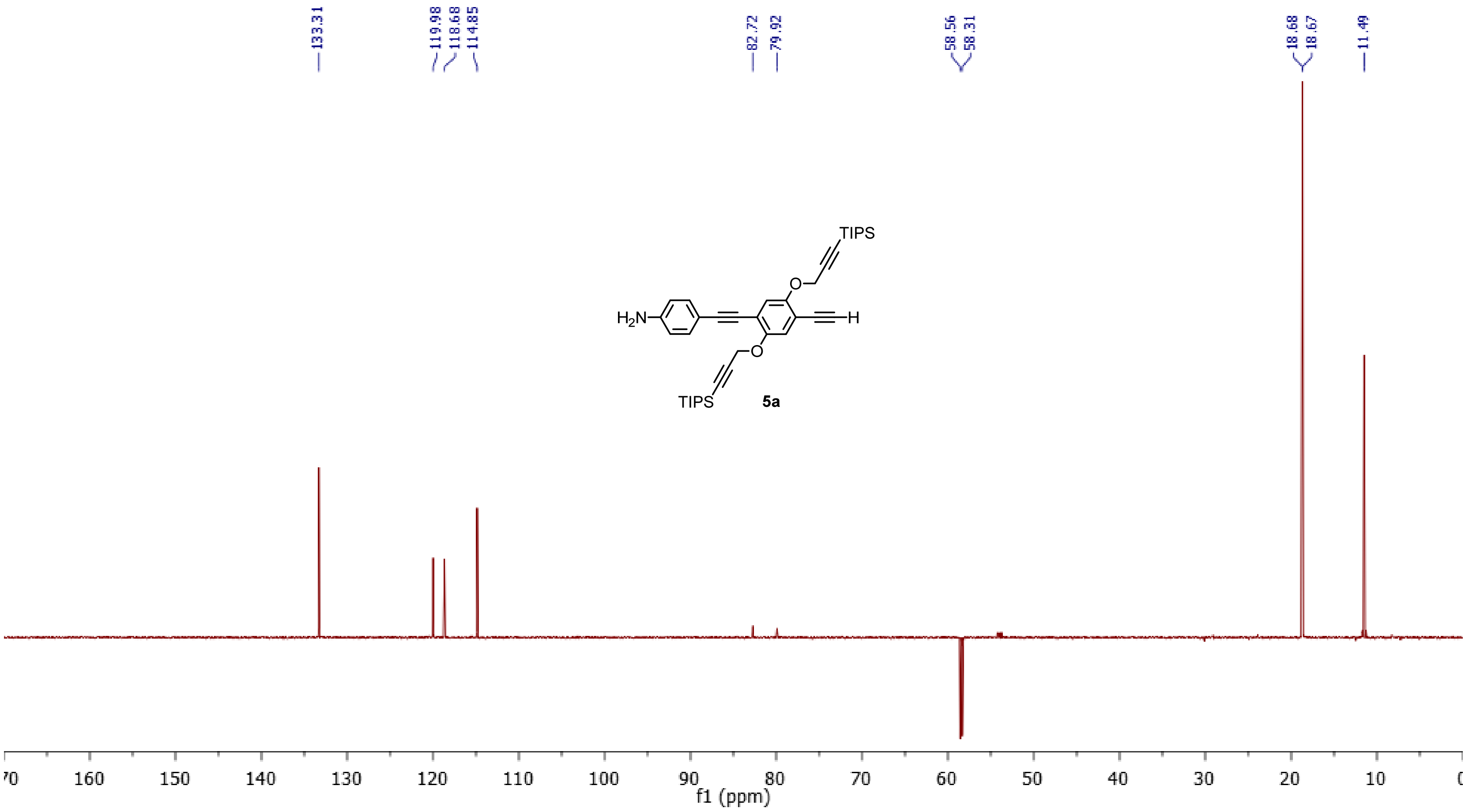

Observed Nucleus: 13C
Observed Frequency: 125.73767221312 MHz

Solvent: CD2Cl2
Number of Scans: 168

Temperature: 296.0974 K
Pulse Sequence: dept135
Relaxation Delay (s): 2 sec

Figure S13. DEPT-135 NMR spectrum of H-(EP)$_2$-NH$_2$ **5a**.

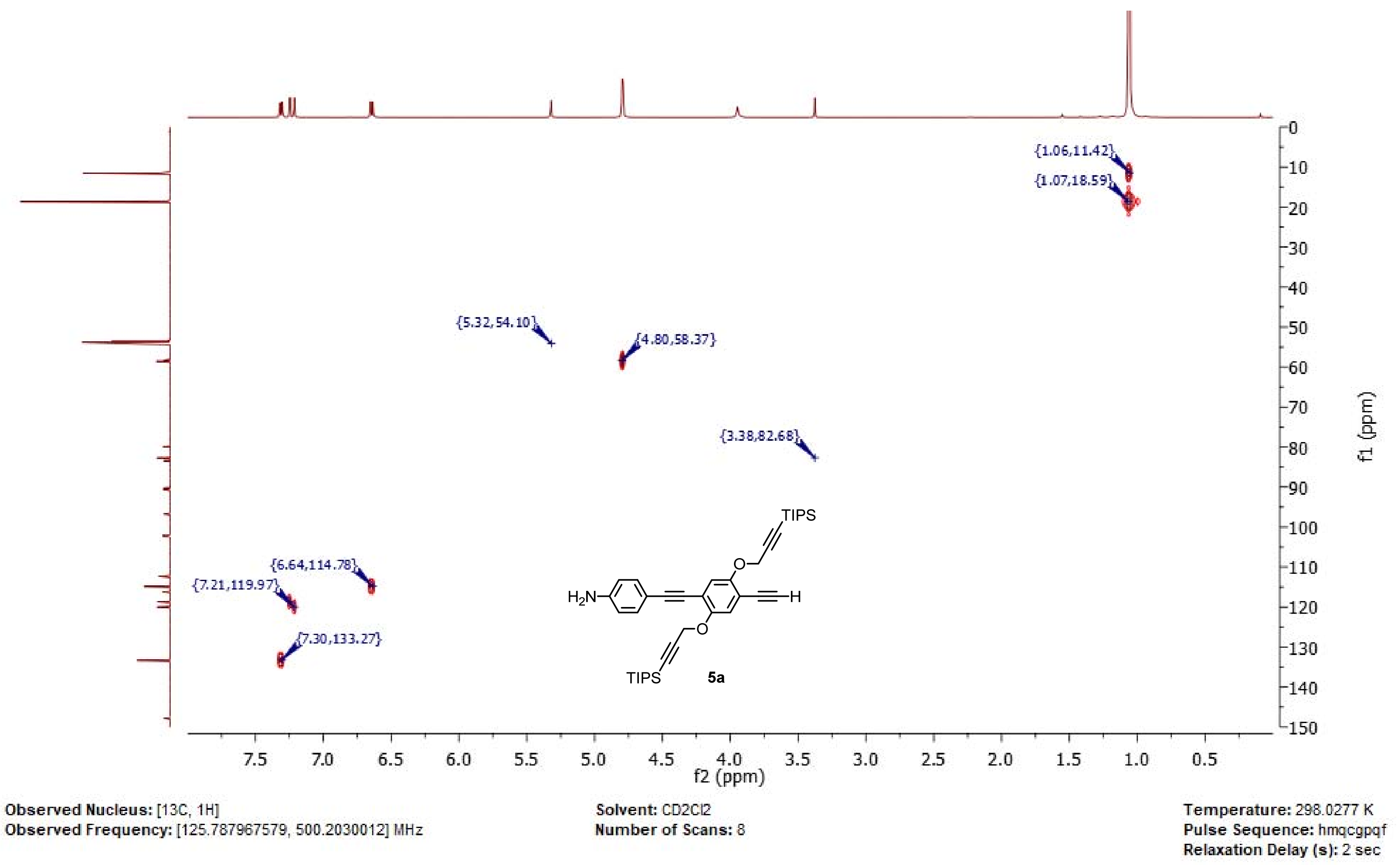

Figure S14. HMQC NMR spectrum of H-$(EP)_2$-NH_2 **5a**.

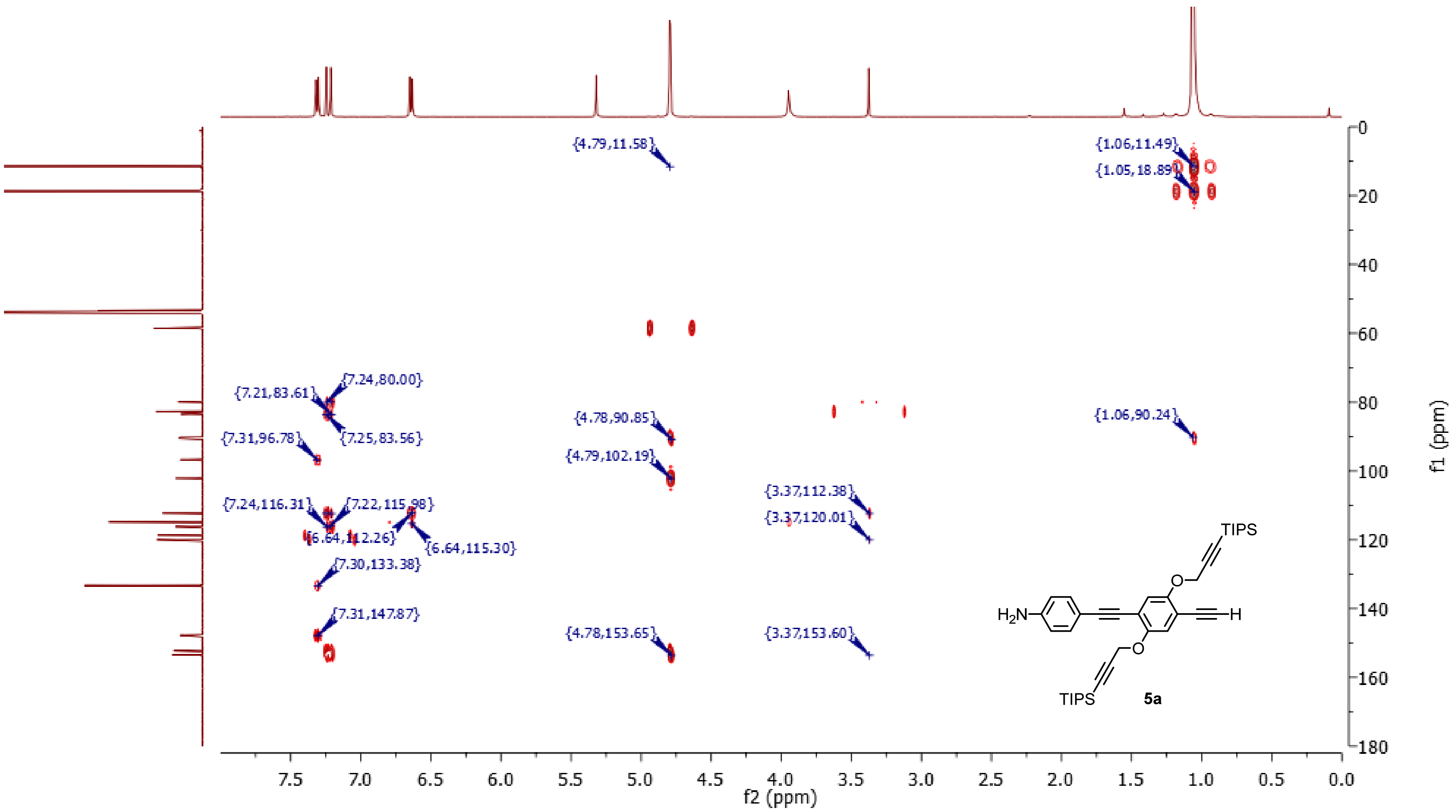

Observed Nucleus: [13C, 1H]
Observed Frequency: [125.787967579, 500.2030012] MHz

Solvent: CD2Cl2
Number of Scans: 8

Temperature: 298.0109 K
Pulse Sequence: hmbcgpndqf
Relaxation Delay (s): 2 sec

Figure S15. HMBC NMR spectrum of H-(EP)$_2$-NH$_2$ **5a**.

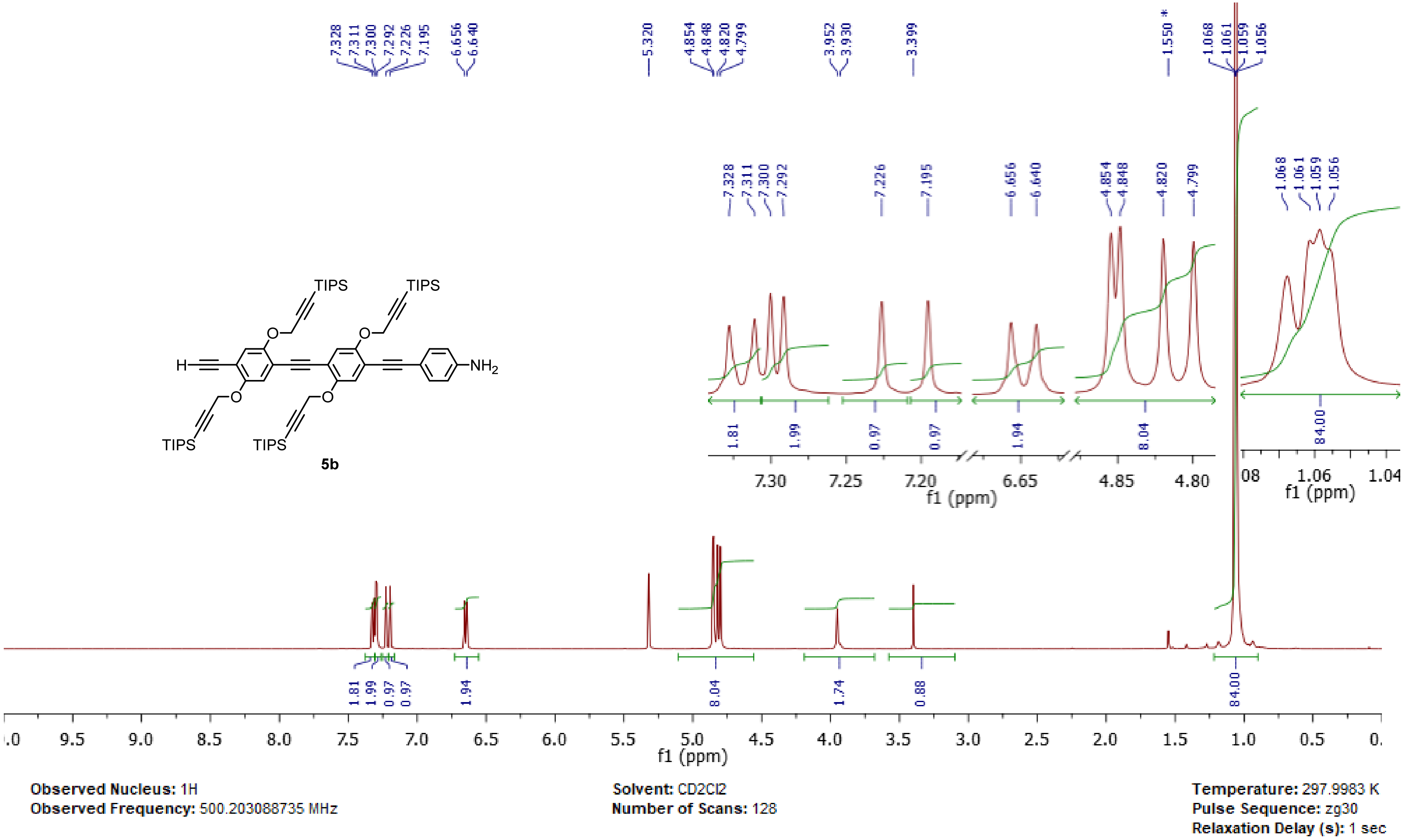

Figure S16. ^{1}H NMR spectrum of H-(EP)$_3$-NH$_2$ **5b**. *H_2O.

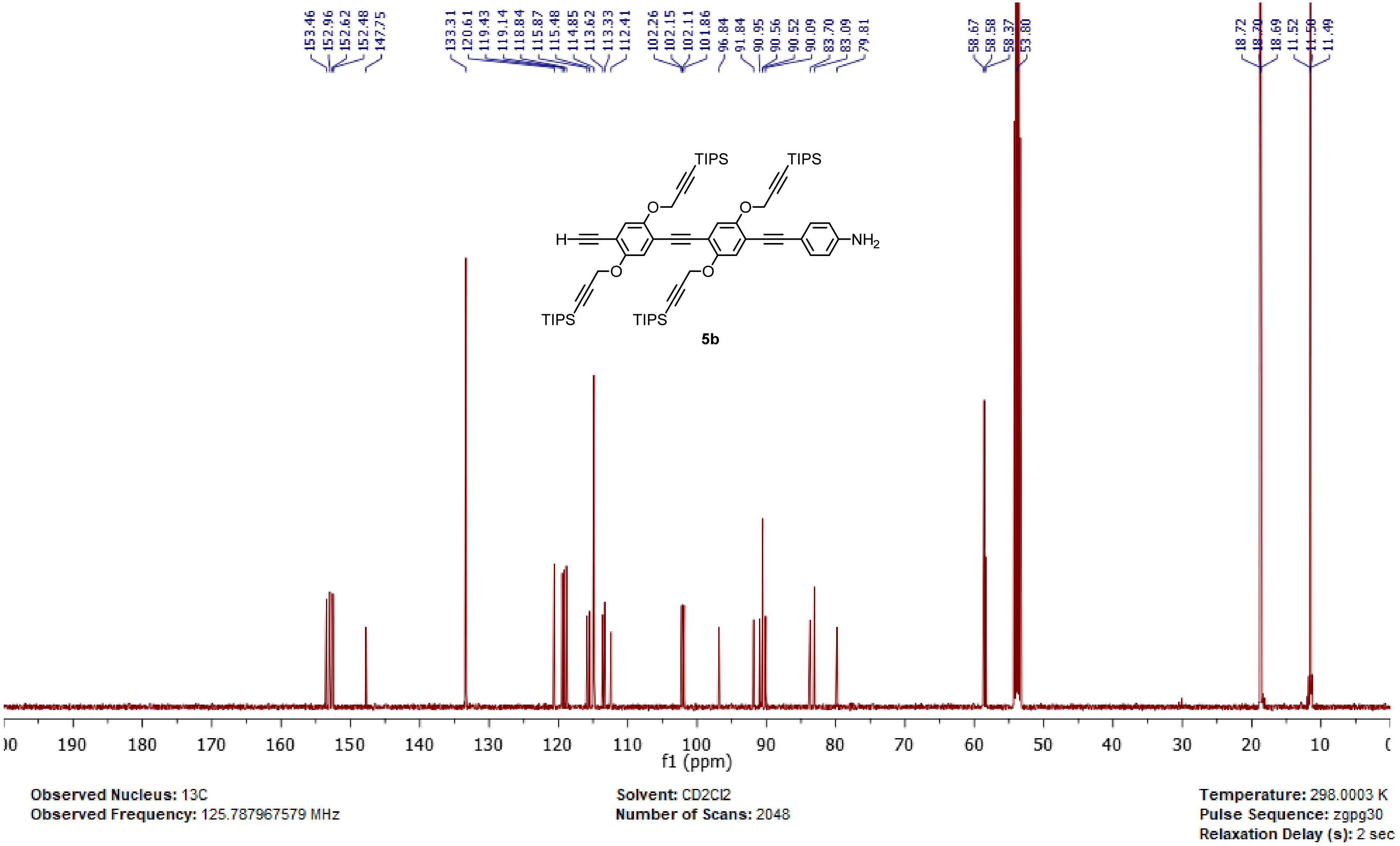

Figure S17. ^{13}C NMR spectrum of H-(EP)$_3$-NH_2 **5b**.

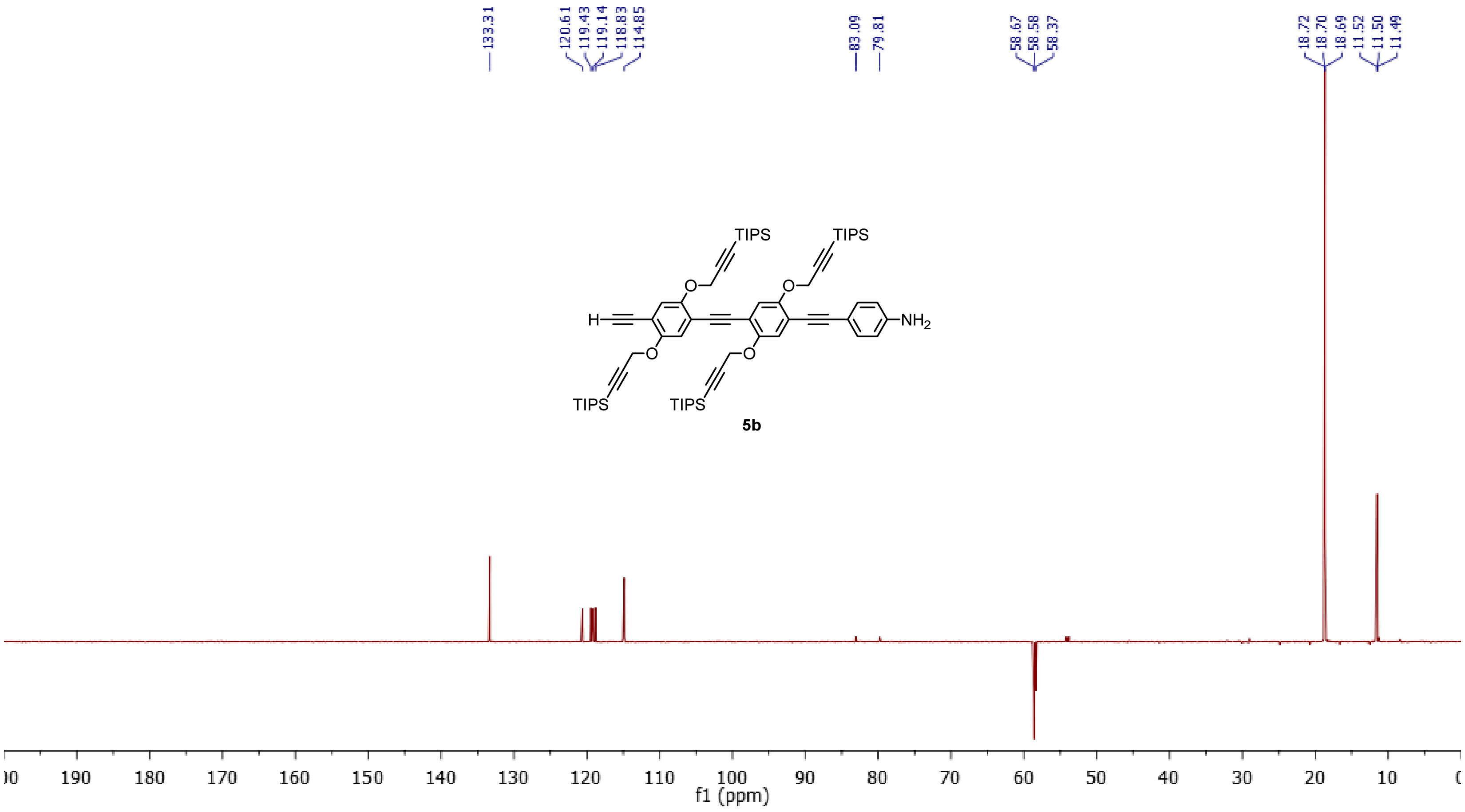

Observed Nucleus: 13C
Observed Frequency: 125.787967579 MHz

Solvent: CD2Cl2
Number of Scans: 512

Temperature: 298.0009 K
Pulse Sequence: deptsp135
Relaxation Delay (s): 2 sec

Figure S18. DEPT-135 NMR spectrum of H-(EP)$_3$-NH$_2$ **5b**.

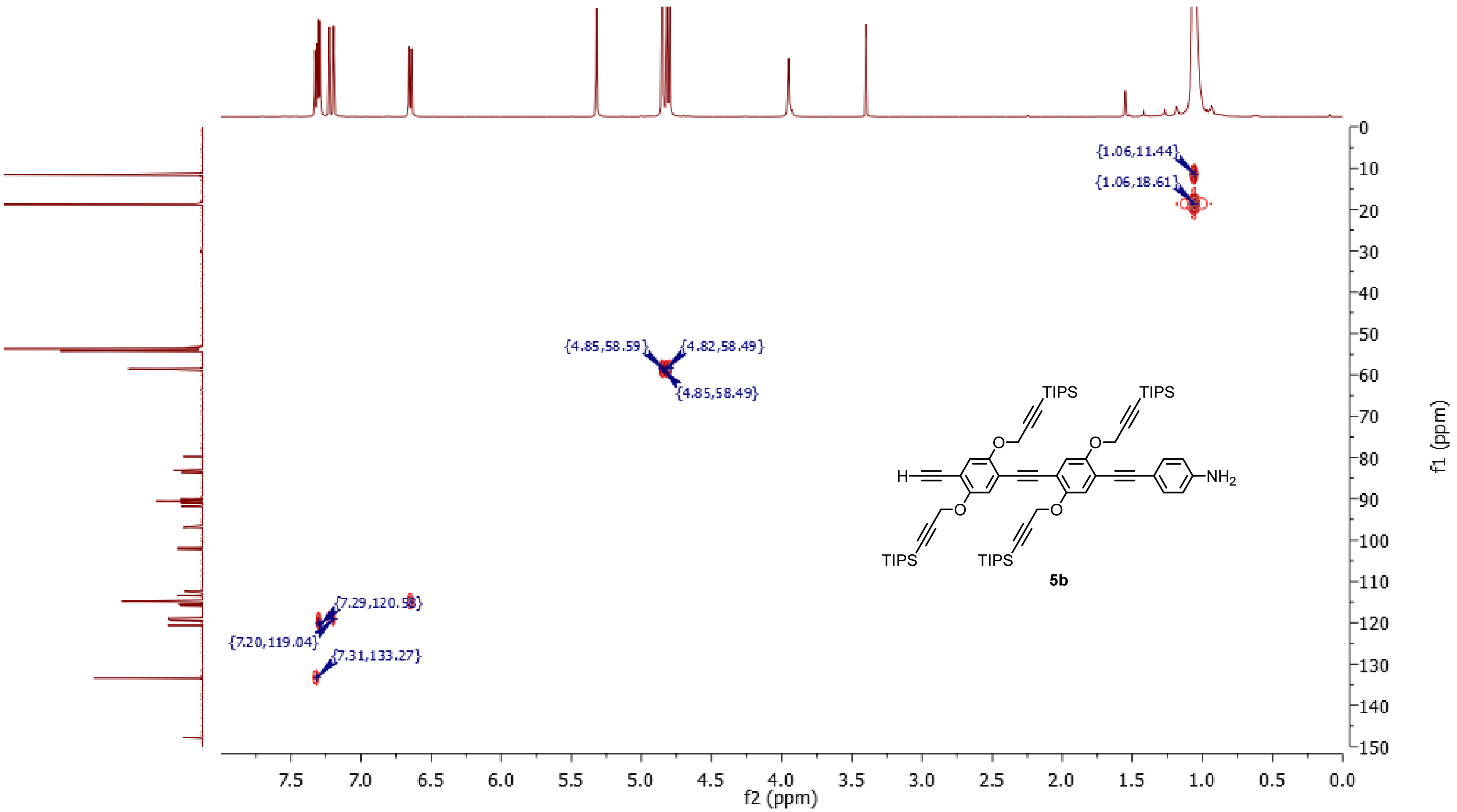

Observed Nucleus: [13C, 1H]
Observed Frequency: [125.787967579, 500.2030012] MHz

Solvent: CD2Cl2
Number of Scans: 8

Temperature: 298.0327 K
Pulse Sequence: hmqcgpqf
Relaxation Delay (s): 2 sec

Figure S19. HMQC NMR spectrum of H-$(EP)_3$-NH_2 **5b**.

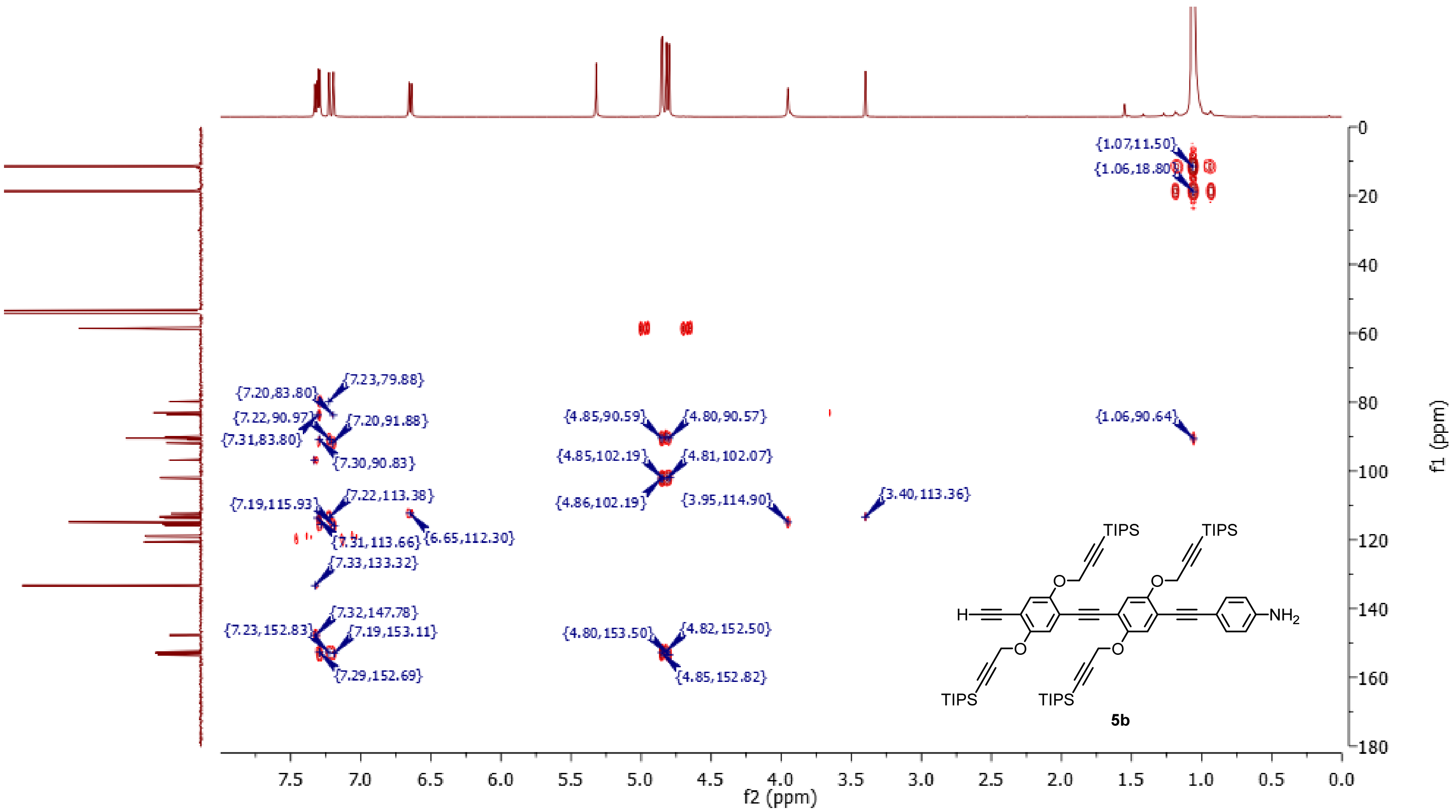

Observed Nucleus: [13C, 1H]
Observed Frequency: [125.787967579, 500.2030012] MHz
Solvent: CD2Cl2
Number of Scans: 8
Temperature: 298.0118 K
Pulse Sequence: hmbcgpndqf
Relaxation Delay (s): 2 sec

Figure S20. HMBC NMR spectrum of H-(EP)$_3$-NH$_2$ **5b**.

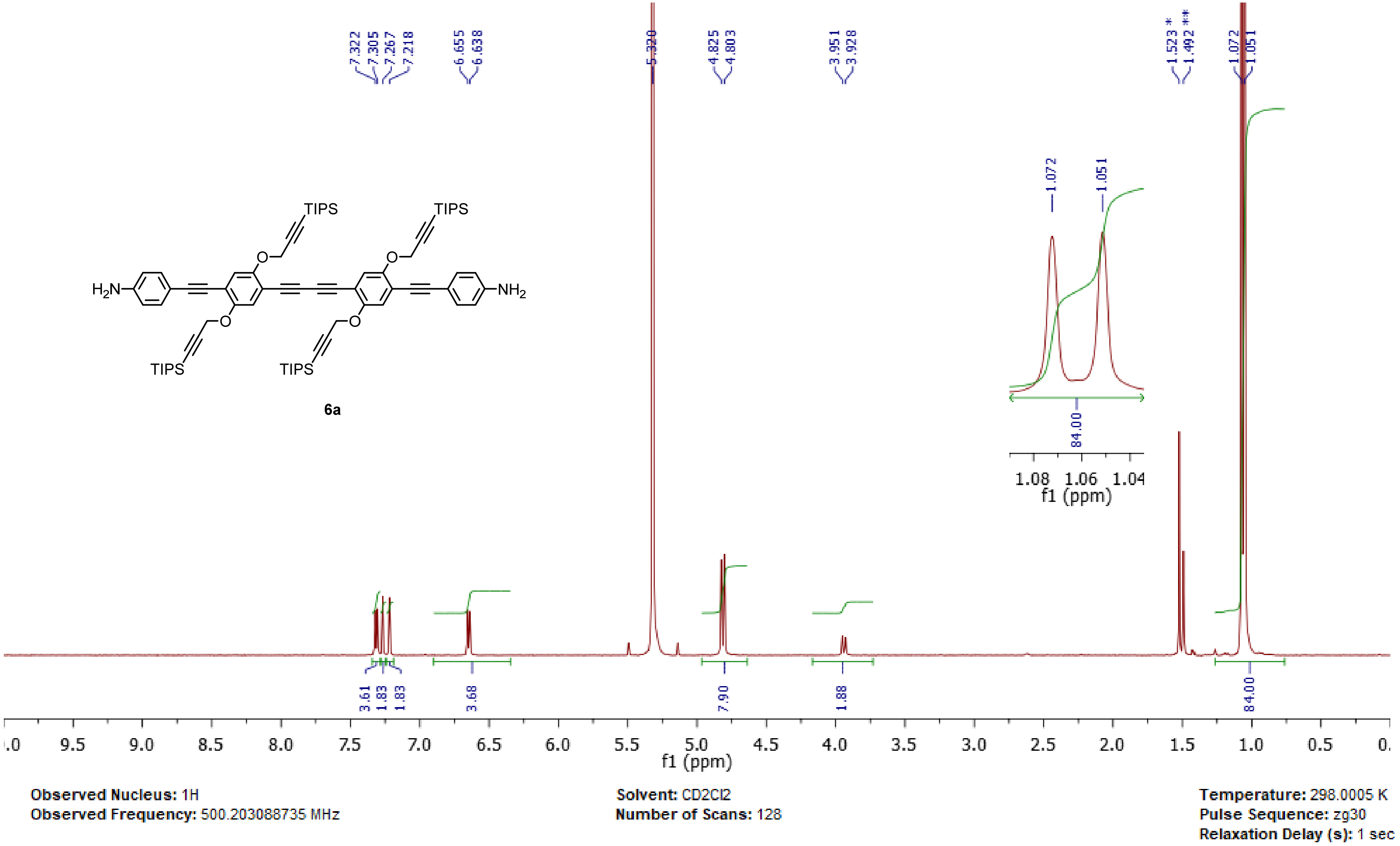

Figure S21. ^1H NMR spectrum of H_2N-PEPBPEP-NH_2 **6a**. *H_2O, **HDO.

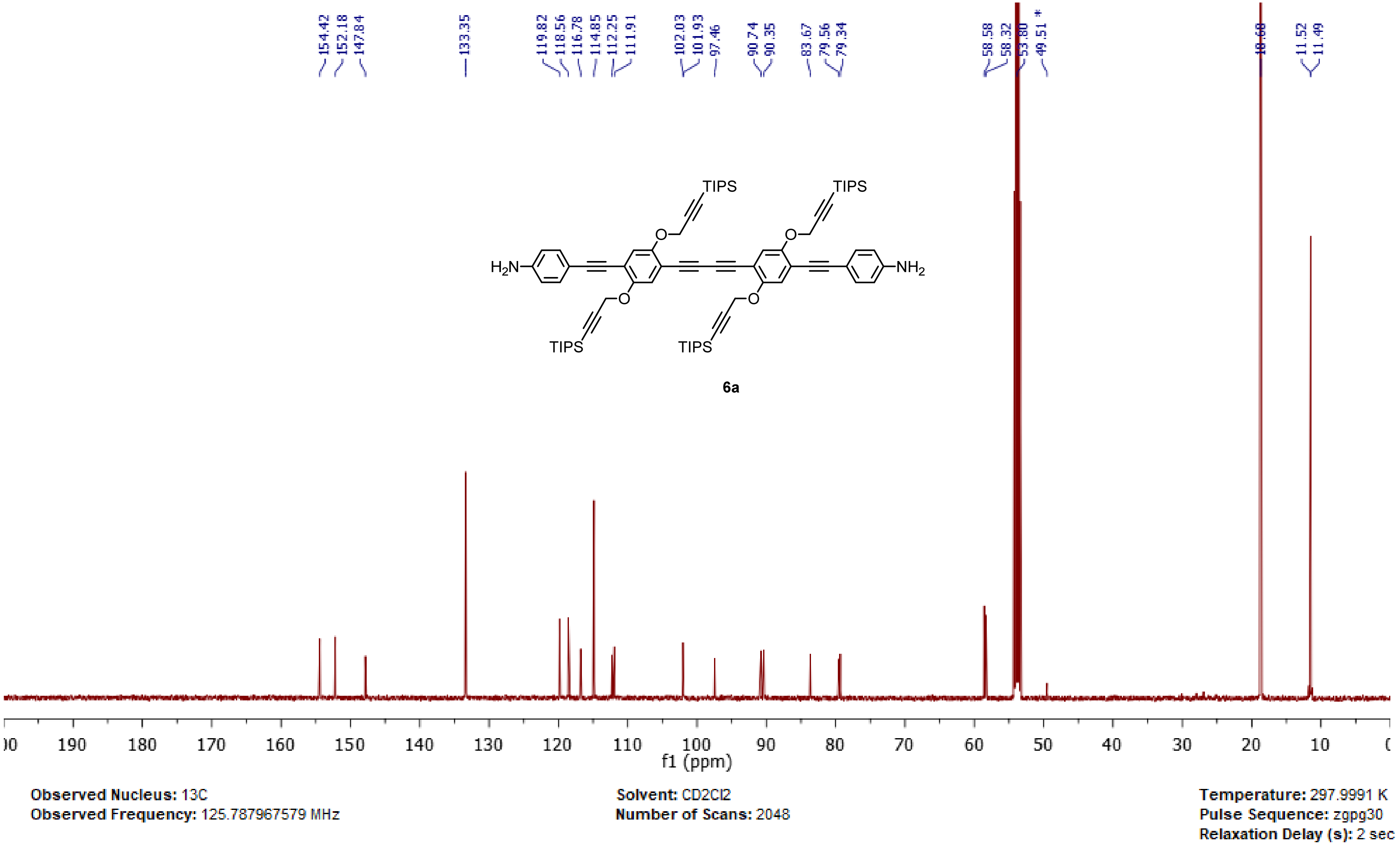

Figure S22. ^{13}C NMR spectrum of H_2N-PEPBPEP-NH_2 **6a**. *Signal belongs to an unidentified compound.

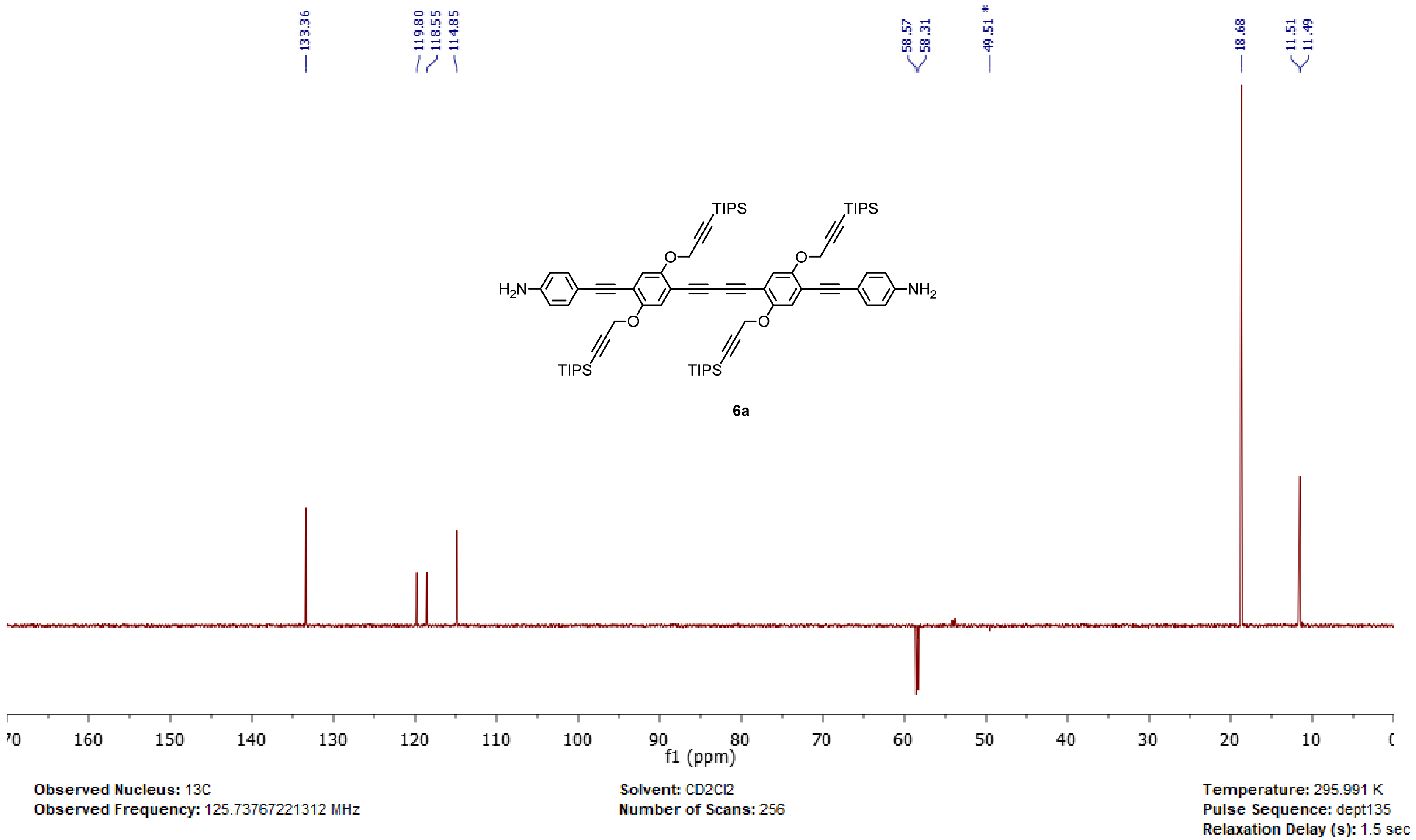

Figure S23. DEPT-135 NMR spectrum of H_2N-PEPBPEP-NH_2 **6a**. *Signal belongs to an unidentified compound.

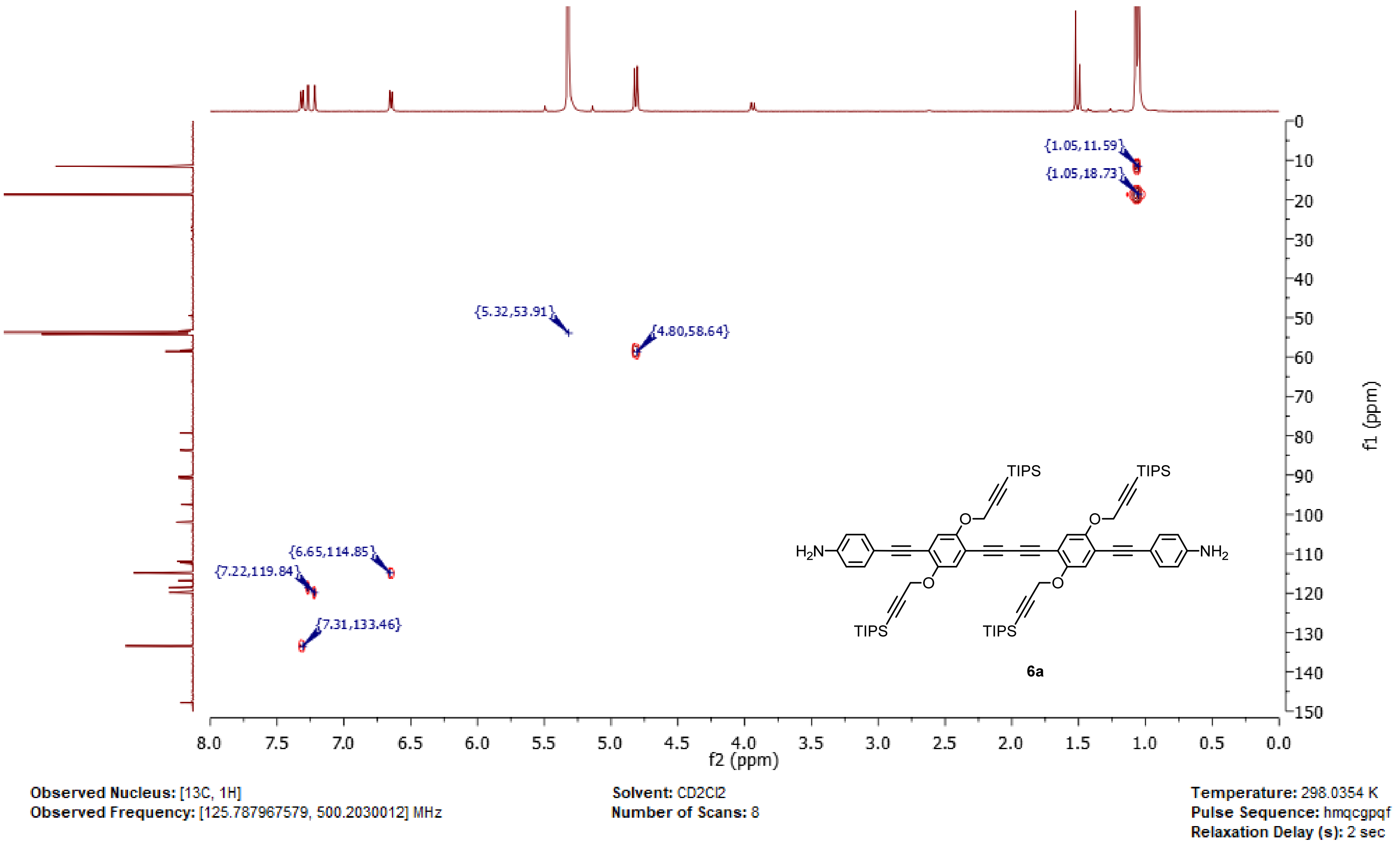

Figure S24. HMQC NMR spectrum of H_2N-PEPBPEP-NH_2 **6a**.

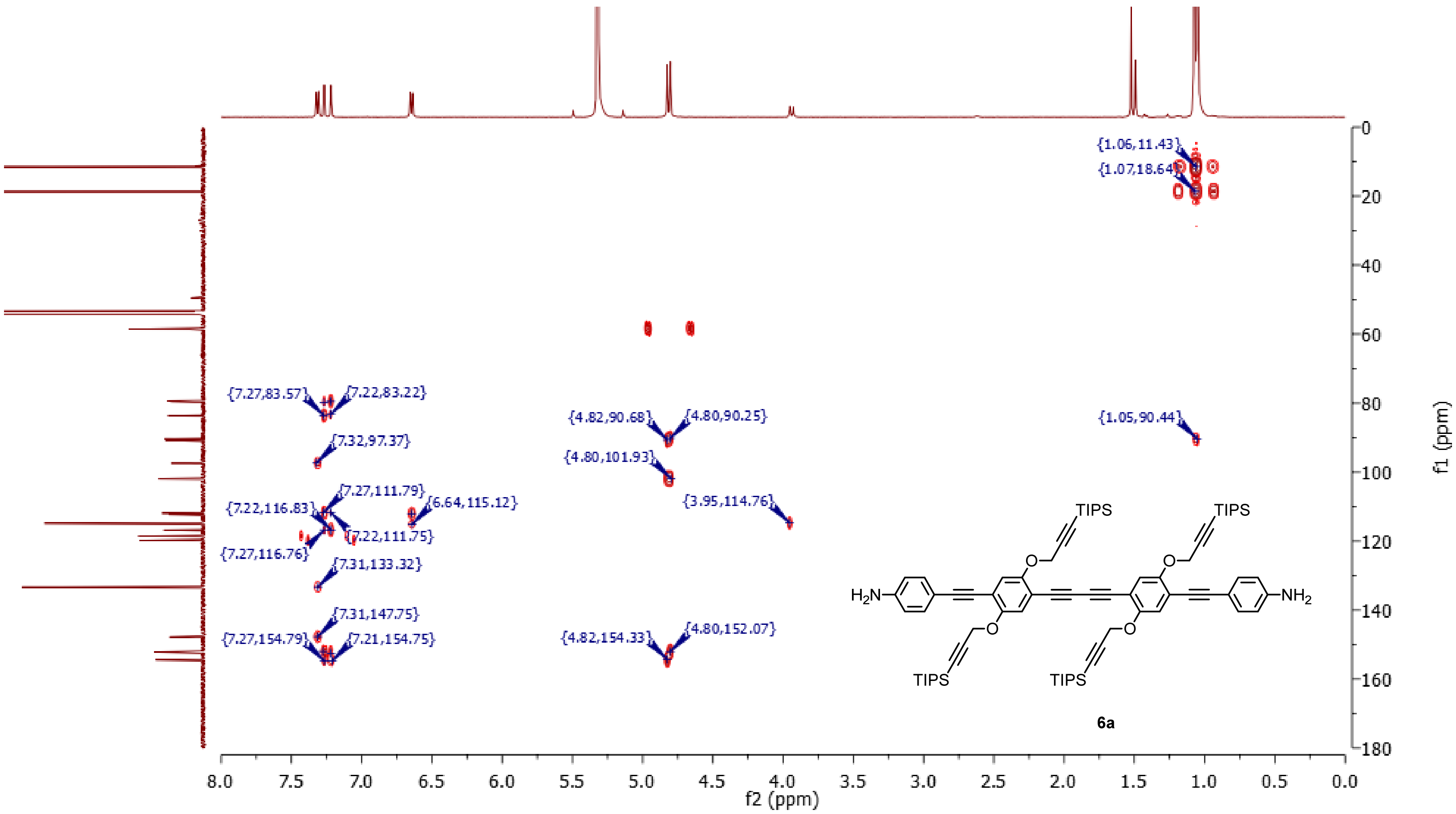

Observed Nucleus: [13C, 1H]
Observed Frequency: [125.787967579, 500.2030012] MHz

Solvent: CD2Cl2
Number of Scans: 8

Temperature: 298.0104 K
Pulse Sequence: hmbcgpndqf
Relaxation Delay (s): 2 sec

Figure S25. HMBC NMR spectrum of H_2N-PEPBPEP-NH_2 **6a**.

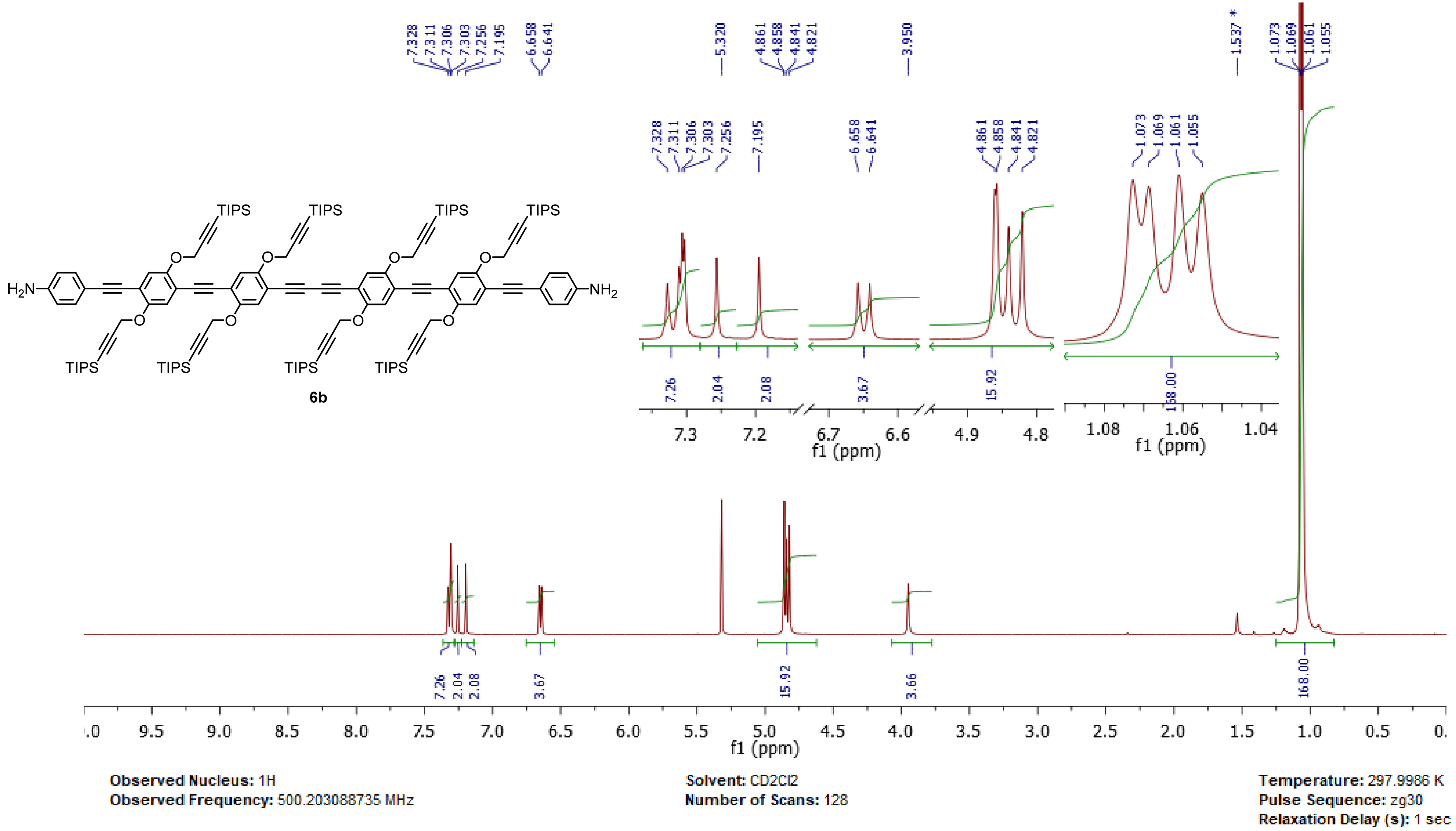

Figure S26. ^{1}H NMR spectrum of H_2N-P(EP)$_2$B(PE)$_2$P-NH_2 **6b**. *H_2O.

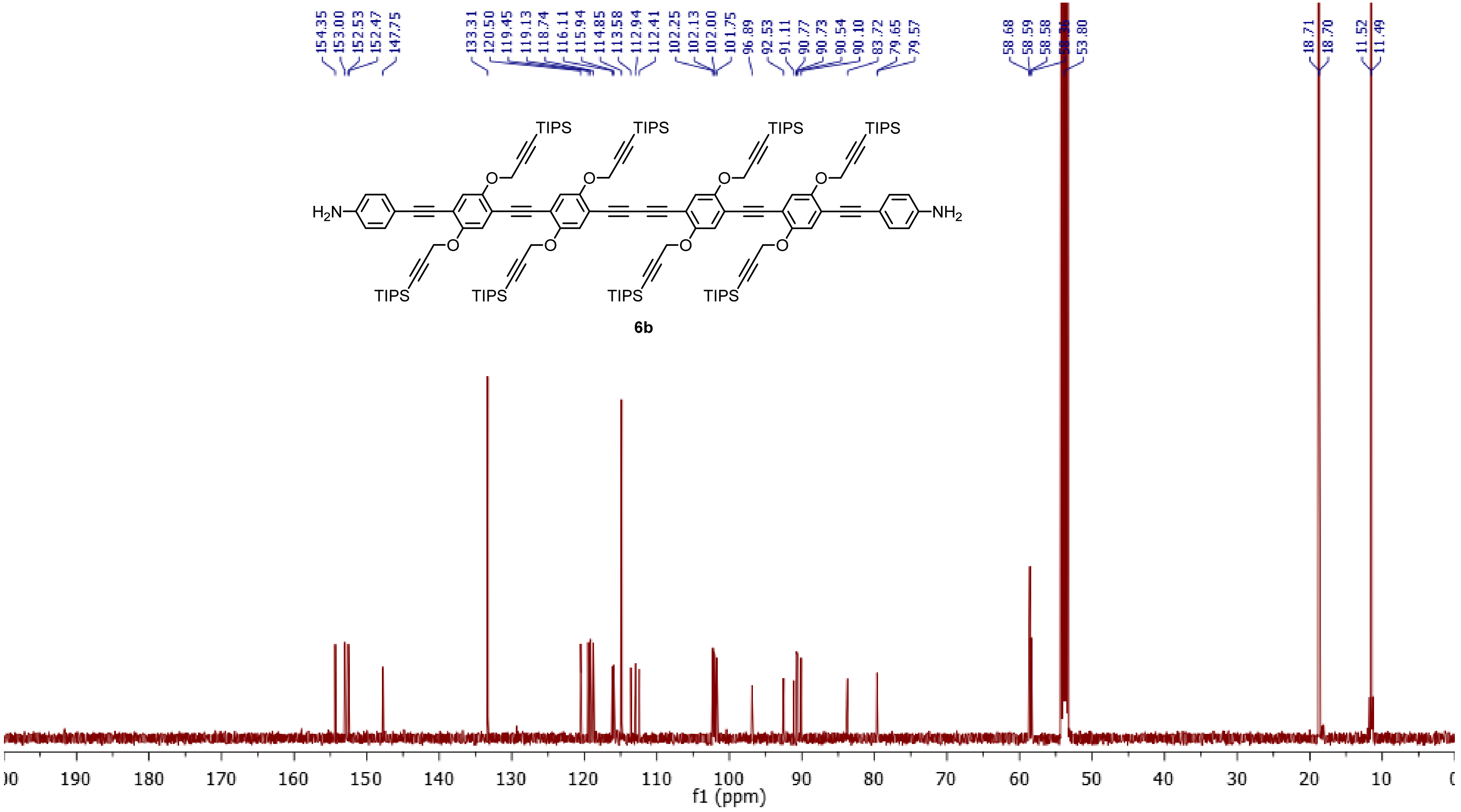

Observed Nucleus: 13C
Observed Frequency: 125.787967579 MHz

Solvent: CD2Cl2
Number of Scans: 2048

Temperature: 297.9941 K
Pulse Sequence: zgpg30
Relaxation Delay (s): 2 sec

Figure S27. ^{13}C NMR spectrum of H_2N-P(EP)$_2$B(PE)$_2$P-NH_2 **6b**.

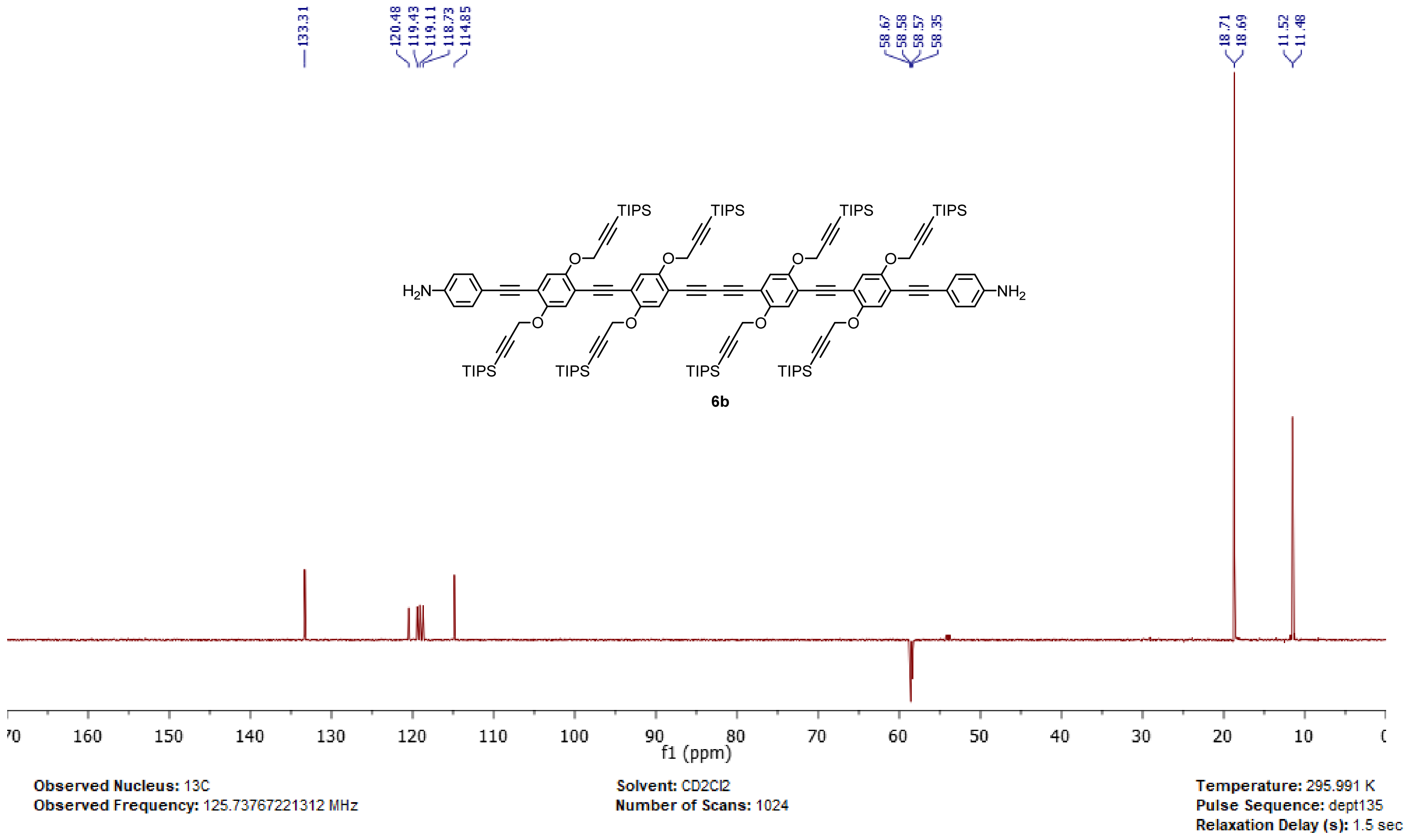

Figure S28. DEPT-135 NMR spectrum of H_2N-P(EP)$_2$B(PE)$_2$P-NH_2 **6b**.

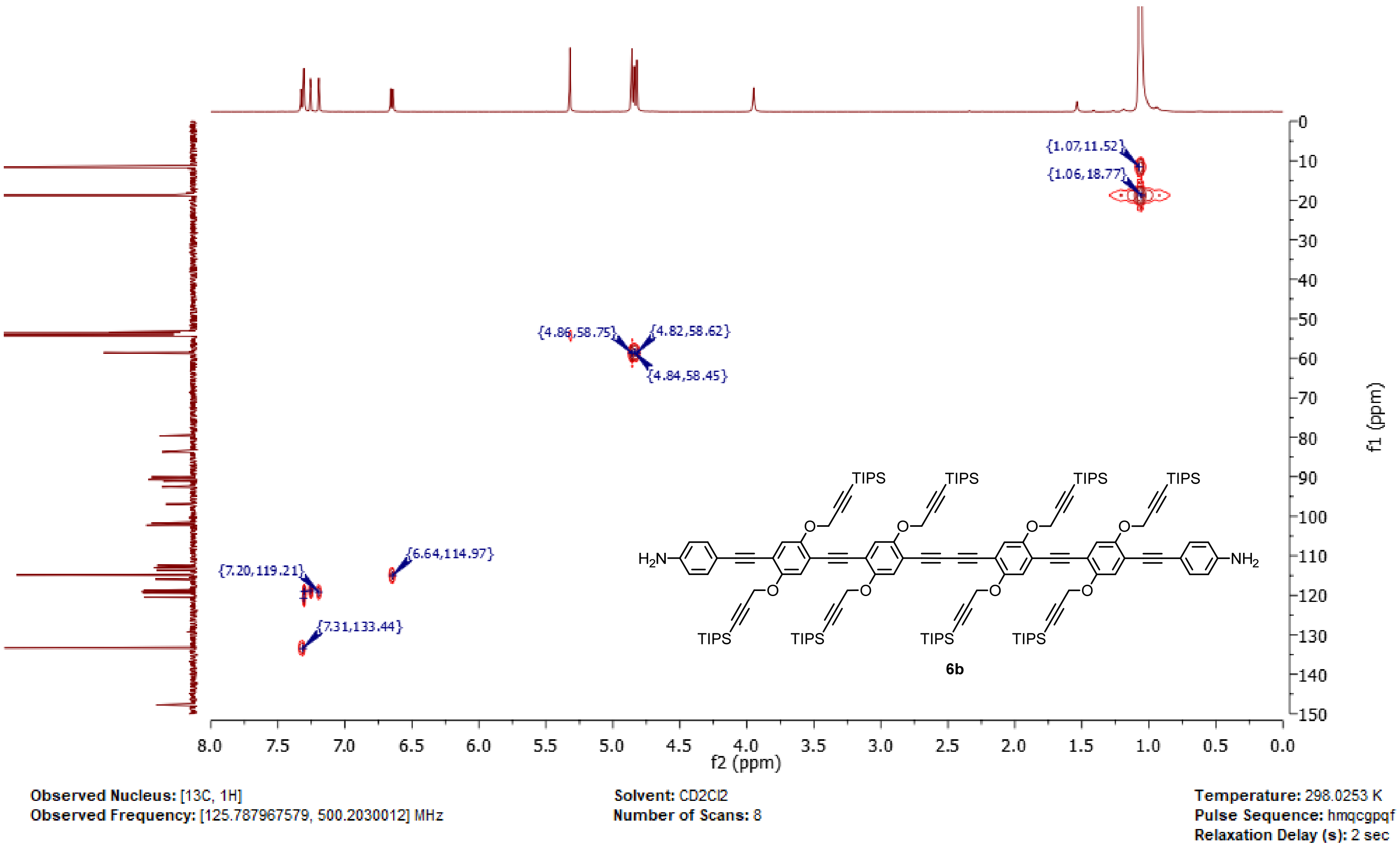

Figure S29. HMQC NMR spectrum of H_2N-P(EP)$_2$B(PE)$_2$P-NH_2 **6b**.

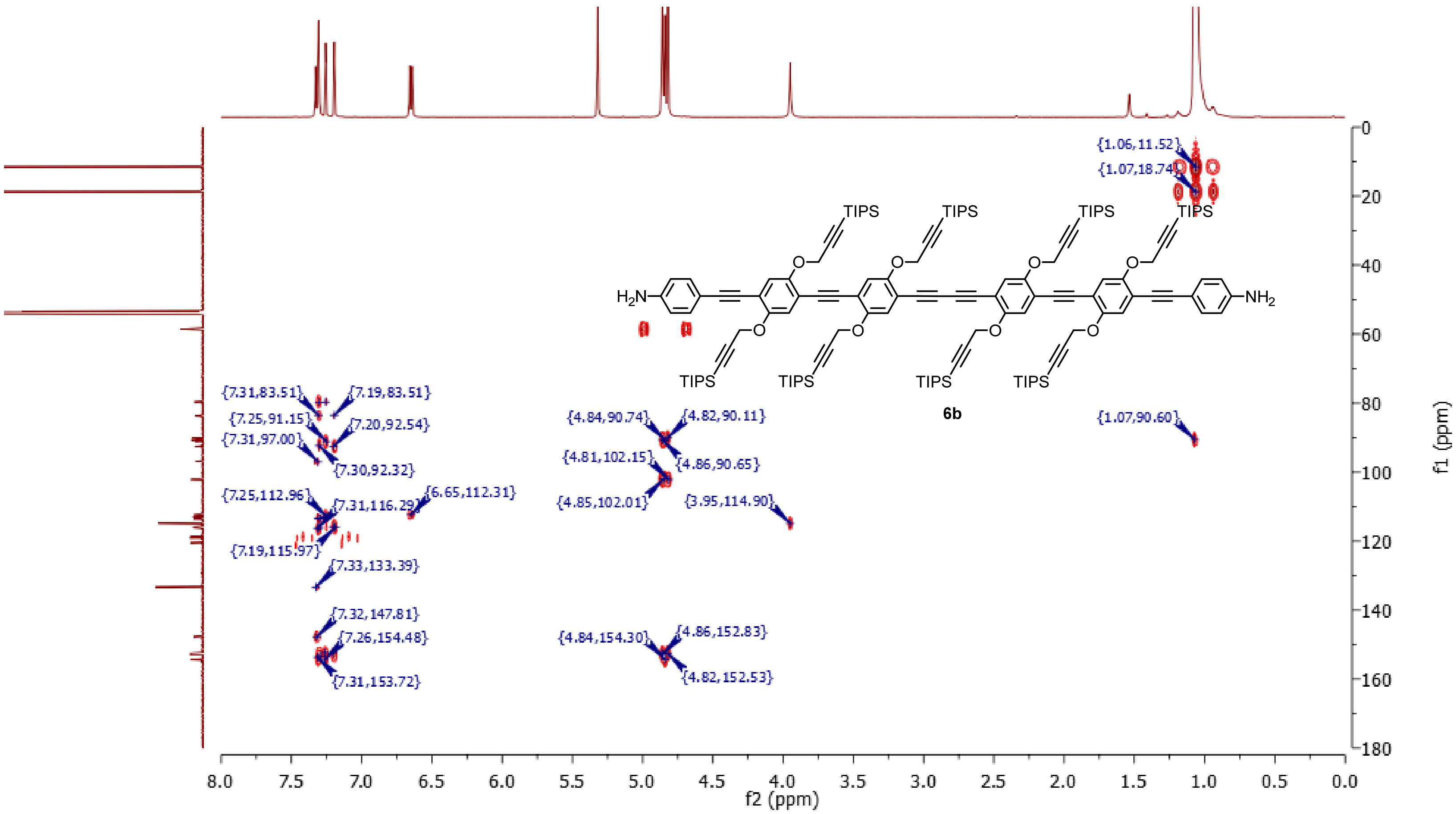

Observed Nucleus: [13C, 1H]
Observed Frequency: [125.787967579, 500.2030012] MHz
Solvent: CD2Cl2
Number of Scans: 16
Temperature: 298.0081 K
Pulse Sequence: hmbcgpndqf
Relaxation Delay (s): 2 sec

Figure S30. HMBC NMR spectrum of H_2N-P(EP)$_2$B(PE)$_2$P-NH_2 **6b**.

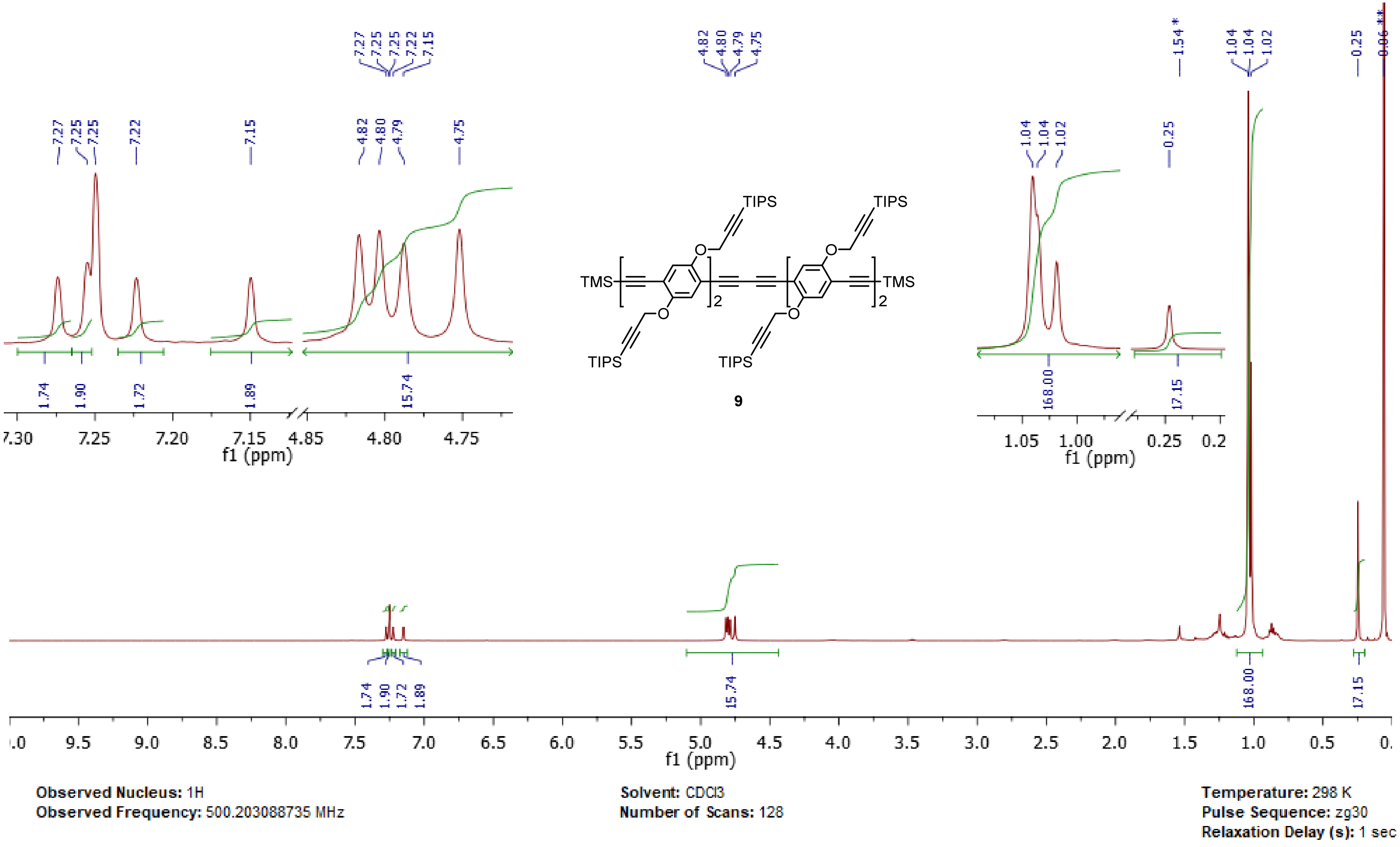

Figure S31. ^{1}H NMR spectrum of alkyne dimer **9**. *H_2O, **grease.
