# Supplementary material for: Distance measurement between trityl radicals by pulse dressed electron paramagnetic resonance with phase modulation
Source: Magn Reson (Gott). 2020 May 15;1(1):75–87. doi: 10.5194/mr-1-75-2020 (PMC10500722; doi:10.5194/mr-1-75-2020)
Supplement: The supplement related to this article is available online at: https://doi.org/10.5194/mr-1-75-2020-supplement. [file mr-1-75-supplement.zip › supplement_revision_1/SI_partA_EPR.pdf]

Supporting Information - Part A
for
Distance measurement between trityl radicals by pulse dressed electron paramagnetic resonance with phase modulation

Nino Wili[1], Henrik Hintz[2], Agathe Vanas[1,‡], Adelheidt Godt[2], and Gunnar Jeschke[2]

[1] *Department of Chemistry and Applied Biosciences, Laboratory of Physical Chemistry, ETH Zurich, Vladimir-Prelog-Weg 2, 8093 Zurich, Switzerland. E-mail: gjeschke@ethz.ch, nwili@ethz.ch*

[1] *Faculty of Chemistry and Center for Molecular Materials (CM_2), Bielefeld University, Universitätsstrasse 25, 33615 Bielefeld, Germany*

Contents

Part A : EPR 2

S.1 Derivation of the nutating frame Hamiltonians 2

S.2 Setting up the dressed spin echo sequence 10

S.3 Implementation details 15

S.4 Additional experimental data 17

S.5 Additional data for figures in the main article 19

S.1 Derivation of the nutating frame Hamiltonians

In this section we will show step-by-step how to derive the effective Hamiltonians in the nutating frame.

S.1.1 Interaction frame transformation

We can split a Hamiltonian $\hat{\mathcal{H}}$ arbitrarily into a dominant part $\hat{\mathcal{H}}_0$ and all the other terms, $\hat{\mathcal{H}}_1$.

$$\hat{\mathcal{H}} = \hat{\mathcal{H}}_0 + \hat{\mathcal{H}}_1 \quad . \tag{1}$$

We can then transform $\hat{\mathcal{H}}$ into an interaction frame with $\hat{\mathcal{H}}_0$ with the unitary transformation

$$\hat{U} = \hat{T} \exp\left(-i \int_0^t \hat{\mathcal{H}}_0(t_1) \mathrm{d}t_1 \right)$$
$$\hat{\mathcal{H}}' = \hat{U}^\dagger \hat{\mathcal{H}} \hat{U} \quad , \tag{2}$$

where $\hat{T}$ is the Dyson time-ordering operator and † indicates the transpose and complex conjugate. For a time-independent $\hat{\mathcal{H}}_0$, the expression simplifies to

$$\hat{U} = \exp\left(-i\hat{\mathcal{H}}_0 t\right) \quad . \tag{3}$$

The equation of motion of the density operator in the interaction frame is then given by

$$\frac{\mathrm{d}}{\mathrm{d}t}\rho' = -i\left[\hat{\mathcal{H}}' - \hat{\mathcal{H}}'_0, \rho'\right] \tag{4}$$
$$\hat{\mathcal{H}}' - \hat{\mathcal{H}}'_0 = \hat{\mathcal{H}}'_1 \tag{5}$$

i.e. the influence of $\hat{\mathcal{H}}_0$ is absorbed into the frame, and we only have to look at the non-dominant terms in the interaction frame. Most of these calculations only involve simple rotations in 3-dimensional space.

S.1.2 Rotating frame

For simplicity and illustration, we first look at an isolated spin-1/2 particle in a static magnetic field B_0 along the laboratory z-axis. If we irradiate this system with a linearly polarized electromagnetic field with frequency ω_{mw} and amplitude $2B_1$, the Hamiltonian in angular frequency units is given by

$$\hat{\mathcal{H}} = \omega_S \hat{S}_z + 2\omega_1 \cos\left(\omega_{\mathrm{mw}} t + \phi_{\mathrm{mw}}\right) \hat{S}_x \quad , \tag{6}$$

We now go into a rotating frame with frequency $\omega_{\rm mw}$. This corresponds to an interaction frame with $\omega_{\rm mw}\hat{S}_z$, *i.e.* $\hat{\mathcal{H}}_0 = \omega_{\rm mw}\hat{S}_z$, which leads to

$$\begin{aligned}\hat{\mathcal{H}}_1' =&\omega_S\hat{S}_z\\ &+2\omega_1\cos\left(\omega_{\rm mw}t+\phi_{\rm mw}\right)\left(\cos(\omega_{\rm mw}t)\hat{S}_x-\sin(\omega_{\rm mw}t)\hat{S}_y\right)\\ &-\omega_{\rm mw}\hat{S}_z\\ =&\Omega_S\hat{S}_z\\ &+\omega_1\left(\cos(\phi_{\rm mw})\hat{S}_x+\sin(\phi_{\rm mw})\hat{S}_y\right)\\ &+\omega_1\left(\cos(2\omega_{\rm mw}t+\phi_{\rm mw})\hat{S}_x-\sin(2\omega_{\rm mw}t+\phi_{\rm mw})\hat{S}_y\right)\quad,\end{aligned}\tag{7}$$

where we used trigonometric identities and introduced the offset $\Omega_S = (\omega_S - \omega_{\rm mw})$. Equation (7) contains two rotating waves, one that rotates with $\omega_{\rm mw}$ that is said to be *on-resonance*, and one that rotates with $-\omega_{\rm mw}$ and is thus far *off*-resonance. The rotating wave approximation (RWA) now simply neglects the counter-rotating component. More formally speaking, this corresponds to the first order average Hamiltonian, obtained by integrating over one modulation period, in this case $\tau_c = 2\pi/\omega_{\rm mw}$,

$$\bar{\hat{\mathcal{H}}}^{(1)} = \frac{1}{\tau_c}\int_0^{\tau_c}\hat{\mathcal{H}}(t_1)\mathrm{d}t_1\quad.\tag{8}$$

Here, the bar indicates the average Hamiltonian, the number in parentheses indicates the order. Quite often, and also in the case of the RWA, this amounts to simply neglecting the remaining time-dependent terms, as they are off-resonant. For the Hamiltonian in Eq. (7) we obtain

$$\hat{\mathcal{H}}_1' \approx \Omega_S\hat{S}_z + \omega_1\left(\cos(\phi_{\rm mw})\hat{S}_x+\sin(\phi_{\rm mw})\hat{S}_y\right)\tag{9}$$

The second order contribution to the average Hamiltonian is given by

$$\bar{\hat{\mathcal{H}}}^{(2)} = \frac{-i}{2\tau_c}\int_0^{\tau_c}\int_0^{t_2}\left[\hat{\mathcal{H}}(t_2),\hat{\mathcal{H}}(t_1)\right]\mathrm{d}t_1\mathrm{d}t_2\quad.\tag{10}$$

Evaluating the double-integral for Eq. (7) leads to the Bloch-Siegert shift Hamiltonian

$$\hat{\mathcal{H}}_{\rm BS} = \frac{1}{4}\frac{\omega_1^2}{\omega_{\rm mw}}\hat{S}_z\quad.\tag{11}$$

S.1.3 Nutating Frame

The truncated rotating frame Hamiltonian in Eq. (9) can be further simplified by assuming $\phi_{\rm mw} = 0$, which leaves us with

$$\hat{\mathcal{H}}' = \Omega_S\hat{S}_z + \omega_1\hat{S}_x\tag{12}$$

If the Rabi frequency is much larger than the resonance offset, $\omega_1 \gg \Omega_S$, the situation can be discussed by using another interaction frame transformation with $\omega_1 \hat{S}_x$. This term is then absorbed into the frame, and the rest transforms to

$$\hat{\mathcal{H}}'' = \Omega_S \left(\cos(\omega_1 t)\hat{S}_z + \sin(\omega_1 t)\hat{S}_y\right) \quad . \tag{13}$$

Here, all terms are time-dependent. Accordingly, the first order average Hamiltonian vanishes

$$\bar{\hat{\mathcal{H}}}''^{(1)} = 0 \quad . \tag{14}$$

It is instructive to look at the second order contribution, which amounts to

$$\bar{\hat{\mathcal{H}}}''^{(2)} = \frac{\Omega_S^2}{2\omega_1}\hat{S}_x \quad . \tag{15}$$

These results can be interpreted in the following way. To a first approximation, the offsets in the rotating frame get averaged out to zero. If this approximation is good, then electrons with different resonance frequencies in the rotating frame have the same resonance frequency in the nutating frame. They become equivalent under the spin-lock. As a second approximation, there is a correction that scales with Ω_S^2/ω_1, and this correction acts along the spin-lock axis of the nutating frame. It depends on the bare-spin resonance offset, Ω_S, and it will thus be different for electrons with different bare-spin resonance frequencies. If the bare-spin resonance offset becomes comparable to the Rabi frequency, it is no longer permissible to neglect higher-order terms, and it is better to use an interaction frame with $\Omega_S \hat{S}_z + \omega_1 \hat{S}_x$. This corresponds to a nutating frame where the axis of rotation is neither along z nor along x, but somewhere in between. However, now the frame transformation depends on the bare-spin resonance frequency, *i.e.*, on a parameter of the spin system rather than only on parameters chosen by the experimenter and being the same for all spins.

S.1.3.1 Hyperfine decoupling and NOVEL

The Hamiltonian for a single electron coupled to a single nucleus in the electron rotating frame is given by

$$\hat{\mathcal{H}}'_{\text{e-n}} = \hat{S}_z \left(A_{xz}\hat{I}_x + A_{yz}\hat{I}_y + A_{zz}\hat{I}_z\right) + \omega_I \hat{I}_z \quad . \tag{16}$$

An interaction frame transformation with $\omega_1 \hat{S}_x$ leads to

$$\begin{aligned}\hat{\mathcal{H}}''_{\text{e-n}} = &\left(\cos(\omega_1 t)\hat{S}_z + \sin(\omega_1 t)\hat{S}_y\right) \\ &\cdot \left(A_{xz}\hat{I}_x + A_{yz}\hat{I}_y + A_{zz}\hat{I}_z\right) + \omega_I \hat{I}_z \quad .\end{aligned} \tag{17}$$

Again, all terms are time-dependent and the first order average Hamiltonian vanishes. If the dominant electron dephasing mechanism relies on the hyperfine coupling, then the dephasing during irradiation should be strongly reduced.

There is, however, a hidden complication, if $\omega_1 \approx \omega_I$. This is best seen by going into the nuclear

rotating frame, *i.e.* the interaction frame with $\omega_I \hat{I}_z$. This leads to

$$\begin{aligned}\hat{\mathcal{H}}''_{\text{e-n}} = &\left(\cos(\omega_1 t)\hat{S}_z + \sin(\omega_1 t)\hat{S}_y\right) \\ &\cdot \left[A_{xz}\left(\cos(\omega_I t)\hat{I}_x - \sin(\omega_I t)\hat{I}_y\right)\right. \\ &+ A_{yz}\left(\cos(\omega_I t)\hat{I}_y + \sin(\omega_I t)\hat{I}_x\right) \\ &\left. + A_{zz}\hat{I}_z\right] \quad . \end{aligned} \tag{18}$$

If ω_1 matches ω_I, some terms proportional to the pseudo-secular couplings, A_{xz} and A_{yz}, become time-independent. Using trigonometric identities and neglecting time-dependent terms, we obtain

$$\begin{aligned}\hat{\mathcal{H}}''_{\text{e-n}} \approx &\frac{1}{2}\left[\hat{S}_y\left(A_{yz}\hat{I}_x - A_{xz}\hat{I}_y\right)\right. \\ &\left. + \hat{S}_z\left(A_{xz}\hat{I}_x + A_{yz}\hat{I}_y\right)\right] \quad . \end{aligned} \tag{19}$$

This Hamiltonian generates nuclear polarization ($\hat{I}_z$) from electron coherence ($\hat{S}_x$) and is thus a mechanism for dynamic nuclear polarization (DNP). The matching condition $\omega_1 = \omega_I$ is called the NOVEL condition[2]. In order to avoid magnetization loss, microwave field strengths close to the NOVEL condition should be avoided unless such polarization transfer is intended.

S.1.3.2 Qualitative discussion of electron dephasing in a nuclear spin bath

Let us look at an electron spin in a bath of nuclei. For simplicity and illustration, we only consider one electron and two nuclei. We assume that the electron is only coupled to nucleus one, we neglect the pseudosecular components of the hyperfine coupling and we do include dipole-dipole coupling between the two nuclei. The Hamiltonian in the electron rotating frame thus reads

$$\hat{\mathcal{H}}' = A_{zz}\hat{S}_z\hat{I}_z + \omega_{12}\left(\hat{I}_{1z}\hat{I}_{2z} - \frac{1}{2}\left(\hat{I}_{1x}\hat{I}_{2x} + \hat{I}_{1y}\hat{I}_{2y}\right)\right) \quad , \tag{20}$$

where ω_{12} is the dipolar coupling constant of the two nuclei (and thus depends on the distance between the nuclei and on their gyromagnetic ratios). Let us assume that we created electron coherence along x' and that it further evolves under the sequence $\tau - (\pi)_y - \tau$. If $\omega_{12} = 0$, the π-pulse completely refocuses the hyperfine coupling, and we end up with $-\hat{S}_{x'}$ after the second delay. However, as the flip-flop terms in the nuclear-nuclear coupling Hamiltonian do not commute with the other terms, for $\omega_{12} \neq 0$ evolution generates two- and three-spin terms of the form $\hat{S}_y\hat{I}_{1x}\hat{I}_{2y}$ (among others). The full expression is rather large and does not provide much insight. However, one easily recognizes that these terms are not refocused by a microwave π-pulse acting on the electron spin. Since the terms are not refocused, the amplitude of the electron coherence must necessarily be reduced. Note that this effect is completely coherent. It does not rely on stochastic flips of the nuclear spins. Extending the argument to a large number of nuclear spins with a distribution of hyperfine and nuclear-nuclear couplings, one expects an apparent decay instead of oscillations. A demonstration by numerical simulations would be computationally expensive, as matrix size grows exponentially with the number of spins. Such computations are beyond the scope of this paper.

Note, however, that a spin-lock that is much stronger than the hyperfine coupling averages said coupling. The nuclear-nuclear coupling can then be neglected because it commutes with all the remaining terms that contain electron operators. In other words, a sufficiently strong spin lock isolates the electron spin from the nuclear spin bath, unless the NOVEL condition is met.

S.1.3.3 Electron-electron coupling in the nutating frame

The dipolar coupling Hamiltonian between two electron spins in the electron rotating frame is given by

$$\hat{\mathcal{H}}'_{\text{e-e,dip}} = \omega_{\text{dd}} \left(\hat{S}_{1z}\hat{S}_{2z} - \frac{1}{2}\left(\hat{S}_{1x}\hat{S}_{2x} + \hat{S}_{1y}\hat{S}_{2y}\right) \right) \quad , \tag{21}$$

as already mentioned in the main text. The situation of strong irradiation of *both* spins is well pictured in a nutating frame obtained by transformation with $\omega_1(\hat{S}_{1x} + \hat{S}_{2x})$. This leads to

$$\begin{aligned} \hat{\mathcal{H}}''_{\text{e-e,dip}} = \omega_{\text{dd}} \big[&(c\hat{S}_{1z} + s\hat{S}_{1y})(c\hat{S}_{2z} + s\hat{S}_{2y}) \\ &-\frac{1}{2}\left(\hat{S}_{1x}\hat{S}_{2x} + (c\hat{S}_{1y} - s\hat{S}_{1z})(c\hat{S}_{2y} - s\hat{S}_{2z})\right)\Big] \end{aligned} \tag{22}$$

with

$$\begin{aligned} c &= \cos(\omega_1 t) \\ s &= \sin(\omega_1 t) \quad . \end{aligned} \tag{23}$$

Using the first order average Hamiltonian approximation and the integrals

$$\frac{1}{\tau_c}\int_0^{\tau_c} c \cdot s\,\mathrm{d}t = 0 \tag{24}$$

$$\frac{1}{\tau_c}\int_0^{\tau_c} c^2\mathrm{d}t = \frac{1}{\tau_c}\int_0^{\tau_c} s^2\mathrm{d}t = 1/2 \tag{25}$$

$$\tag{26}$$

we obtain the result shown in the main text

$$\begin{aligned} \hat{\mathcal{H}}''_{\text{e-e,dip}} \approx \omega_{\text{dd}} \Big[&\frac{1}{2}\hat{S}_{1z}\hat{S}_{2z} + \frac{1}{2}\hat{S}_{1y}\hat{S}_{2y} \\ &-\frac{1}{2}\left(\hat{S}_{1x}\hat{S}_{2x} + \frac{1}{2}\hat{S}_{1y}\hat{S}_{2y} + \frac{1}{2}\hat{S}_{1z}\hat{S}_{2z}\right)\Big] \\ = -\frac{\omega_{\text{dd}}}{2} &\left[\hat{S}_{1x}\hat{S}_{2x} - \frac{1}{2}\left(\hat{S}_{1z}\hat{S}_{2z} + \hat{S}_{1y}\hat{S}_{2y}\right)\right] \end{aligned} \tag{27}$$

Note that the simplification leading to this result crucially depends on the condition that both spins are irradiated with the same strength and that the irradiation is stronger than the difference in rotating frame offsets. If the difference in offsets is larger than the dipolar coupling or the Rabi frequency,

$\Delta\Omega > \omega_1, \omega_{\mathrm{dd}}$, then the pseudosecular terms in Eq. (21) are already averaged in the doubly rotating frame (meaning that the frames for the two electrons rotate with different frequencies). If only one spin is irradiated, the average dipole-dipole coupling Hamiltonian will vanish. However, if the two spins were irradiated separately with two different frequencies and the same Rabi frequency, part of the Hamiltonian would again be time-independent. This is a situation very similar to Hartmann-Hahn cross-polarization in solid-state NMR.

Regarding the exchange coupling, one can simply argue that it involves a scalar product of spin operators. The scalar product is invariant under frame transformations. However, this statement is slightly dubious because the scalar product of spin operators connects two different spin spaces and may no longer be invariant if the transformations of the two frames differ. Therefore, depending on the irradiation scheme the exchange coupling is not trivially the same between dressed spins as between bare spins. Here, we show explicitly that the exchange coupling is invariant if the same frame transformation is applied to both spins. For an exchange coupling Hamiltonian given by

$$\begin{aligned}\hat{\mathcal{H}}'_{\text{e-e,J}} &= J\left(\hat{\vec{S}}_1 \cdot \hat{\vec{S}}_2\right) \\ &= J\left(\hat{S}_{1x}\hat{S}_{2x} + \hat{S}_{1y'}\hat{S}_y + \hat{S}_{1z}\hat{S}_{2z}\right) \quad ,\end{aligned} \tag{28}$$

we employ an interaction frame transformation with $\omega_1(\hat{S}_{1x} + \hat{S}_{2x})$, leading to

$$\begin{aligned}\hat{\mathcal{H}}''_{\text{e-e,J}} &= J\,(\hat{S}_{1x}\hat{S}_{2x} \\ &\qquad + (c\hat{S}_{1y} - s\hat{S}_{1z})(c\hat{S}_{2y} - s\hat{S}_{2z}) \\ &\qquad + (c\hat{S}_{1z} + s\hat{S}_{1y})(c\hat{S}_{2z} + s\hat{S}_{2y})) \\ &= J\left(\hat{S}_{1x}\hat{S}_{2x} + \hat{S}_{1y}\hat{S}_{2y} + \hat{S}_{1z}\hat{S}_{2z}\right) \quad .\end{aligned} \tag{29}$$

which is the same as before the interaction frame transformation.

S.1.3.4 Details on the dressed spin echo

Here we explicitly show some more details regarding the dressed spin echo sequence. We assume that the electron spins as well as the spin-lock are along the x' axis. The density operator in the usual rotating frame is then described by

$$\hat{\sigma}'_1 = \hat{S}_{1x} + \hat{S}_{2x} \quad . \tag{30}$$

The Hamiltonian describing the microwave irradiation in the rotating frame is given by

$$\hat{\mathcal{H}}'_{\mathrm{mw}} = \omega_1\left(\cos\left(\phi_{\mathrm{mw}}(t)\right)\hat{F}_x + \sin\left(\phi_{\mathrm{mw}}(t)\right)\hat{F}_y\right) \quad , \tag{31}$$

with $\hat{F}_x = \hat{S}_{1x} + \hat{S}_{2x}$. The above expression, in the absence of any phase-modulation ($\phi_{\mathrm{mw}}(t) = 0$), reduces simply to

$$\hat{\mathcal{H}}'_{\mathrm{mw}} = \omega_1\hat{F}_x \quad . \tag{32}$$

As long as the spins and the spin-lock field point along the same direction, nothing happens (apart from relaxation, which is not treated in this formalism). This is even true if we include the dipolar coupling, because again, the sum of polarization does not evolve under the dipolar coupling Hamiltonian.

In order to describe a phase pulse in the rotating frame, we would need to use the Hamiltonian in Eq. (31). The corresponding propagator is given by

$$\hat{U} = \hat{T} \exp\left(-i \int_0^{t_p} \hat{\mathcal{H}}'_{\text{mw}}(t) \mathrm{d}t\right) \tag{33}$$

where $\hat{T}$ is the Dyson time ordering operator. This integral is more of a formality, because it is usually not evaluated this way. Numerically, one can calculate the propagator by time-slicing, *i.e.* by evaluating the time-dependent Hamiltonian and the corresponding propagator for short time steps and calculating the integral by multiplying propagators of the small time steps in the correct order. However, it is much easier to describe a phase pulse in the nutating frame! In this frame, the density matrix above is still given by

$$\hat{\sigma}''_1 = \hat{S}_{1x} + \hat{S}_{2x} \quad , \tag{34}$$

but the Hamiltonian, after the rotating wave approximation, reduces to

$$\hat{\mathcal{H}}''_{\text{mw}} = \frac{\omega_1 a_{\text{PM}}}{2} \left(\cos\left(\phi_{\text{PM}}\right) \hat{F}_y + \sin\left(\phi_{\text{PM}}\right) \hat{F}_z\right) \quad , \tag{35}$$

which for $\phi_{\text{PM}} = \pi$ (arbitrary) reduces to

$$\hat{\mathcal{H}}''_{\text{mw}} = -\frac{\omega_1 a_{\text{PM}}}{2} \hat{F}_y \quad . \tag{36}$$

This expression is time-independent. The corresponding propagator is then given by

$$\hat{U} = \exp\left(i \frac{\omega_1 a_{\text{PM}}}{2} \hat{F}_y t_p\right) \quad . \tag{37}$$

If we choose $\frac{\omega_1 a_{\text{PM}}}{2} \cdot t_p = \pi/2$, we obtain

$$\hat{U} = \exp\left(i \frac{\pi}{2} \hat{F}_y\right) \quad , \tag{38}$$

which corresponds to a $\frac{pi}{2}$ pulse in the nutating frame. Applied to the density matrix before the pulse, we get

$$\hat{\sigma}''_2 = \hat{U} \hat{\sigma}''_1 \hat{U}^\dagger = \hat{S}_{1z} + \hat{S}_{2z} \quad . \tag{39}$$

The last line can be represented in the product operator formalism (POF) by

$$\hat{S}_{1x} + \hat{S}_{2x} \xrightarrow{-\frac{\pi}{2} \hat{F}_y} \left(\hat{S}_{1z} + \hat{S}_{2z}\right) \tag{40}$$

Now we explicitly show how this state evolves under the dipolar Hamiltonian in the nutating

frame, *i.e.*

$$\hat{S}_{1z} + \hat{S}_{2z} \xrightarrow{-\frac{\omega_{\mathrm{dd}}}{2}\left(\hat{S}_{1x}\hat{S}_{2x} - \frac{1}{2}\left(\hat{S}_{1z}\hat{S}_{2z} + \hat{S}_{1y}\hat{S}_{2y}\right)\right)\cdot t} ? \tag{41}$$

It is important to note that all the two-spin operators in the Hamiltonian commute with each other. This means that we can evaluate their influence on the density operator step-by-step in the POF. We also do it for one spin only and then obtain the result of the second spin by changing the corresponding indices.

$$\begin{aligned}
\hat{S}_{1z} &\xrightarrow{-\frac{\omega_{\mathrm{dd}}}{2}\left(\hat{S}_{1x}\hat{S}_{2x}\right)\cdot t} \cos(\frac{\omega_{\mathrm{dd}}}{4}t)\hat{S}_{1z} + \sin(\frac{\omega_{\mathrm{dd}}}{4}t)\hat{S}_{1y}\hat{S}_{2x} \\
&\xrightarrow{+\frac{\omega_{\mathrm{dd}}}{4}\left(\hat{S}_{1y}\hat{S}_{2y} + \hat{S}_{1z}\hat{S}_{2z}\right)\cdot t} \cos(\frac{\omega_{\mathrm{dd}}}{4}t)\left[\cos(\frac{\omega_{\mathrm{dd}}}{8}t)\hat{S}_{1z} + \sin(\frac{\omega_{\mathrm{dd}}}{8}t)\hat{S}_{1x}\hat{S}_{2z}\right] \\
&\qquad + \sin(\frac{\omega_{\mathrm{dd}}}{4}t)\left[\cos(\frac{\omega_{\mathrm{dd}}}{8}t)\hat{S}_{1y}\hat{S}_{2x} - \sin(\frac{\omega_{\mathrm{dd}}}{8}t)\hat{S}_{2z}\right]
\end{aligned} \tag{42}$$

Including the result for the second spin, and only keeping terms proportional to $\hat{S}_{1z} + \hat{S}_{2z}$ gives the signal

$$\begin{aligned}
s(t) &= \cos(\frac{\omega_{\mathrm{dd}}}{4}t)\cos(\frac{\omega_{\mathrm{dd}}}{8}t) - \sin(\frac{\omega_{\mathrm{dd}}}{4}t)\sin(\frac{\omega_{\mathrm{dd}}}{8}t) \\
&= \frac{1}{2}\left(\cos(\frac{3}{8}\omega_{\mathrm{dd}}t) + \cos(\frac{1}{8}\omega_{\mathrm{dd}}t) + \cos(\frac{3}{8}\omega_{\mathrm{dd}}t) - \cos(\frac{1}{8}\omega_{\mathrm{dd}}t\right) \\
&= \cos(\frac{3}{8}\omega_{\mathrm{dd}}t)
\end{aligned} \tag{43}$$

Note that in the main text, we display the echo intensity as a function of τ_1. The effective dipolar evolution time is $2 \cdot \tau_1$, leading to

$$s(t) = \cos(\frac{3}{4}\omega_{\mathrm{dd}}\tau_1) \tag{44}$$

S.2 Setting up the dressed spin echo sequence

Here we give a detailed account of the measurement setup for dressed pulse sequences. We assume that the sample is in the spectrometer at the correct temperature and field and that you are able to get an echo.

1. **Set up $\pi/2$ and π pulse lengths** by maximizing the echo amplitude of a Hahn echo where the second pulse is twice as long as the first one. This step seems trivial, but it already suffers from some complications in the case of dipolar coupled trityls, which are seen in the following Figure S1. Clearly, there are two close lying local maxima, which is not compatible with the behavior of a simple spin-1/2 system. The local maxima are due to the small ratio of spectral width compared to the dipolar coupling, and the exact curve depends on the value of the interpulse delay. Note that for the dressed spin sequences, one generally prefers to choose this interpulse delay for the readout as short as possible. A similar observation was reported by Meyer *et al.*[3]

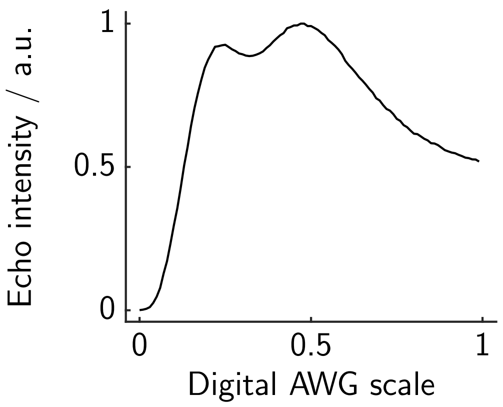

Fig. S1: Echo intensity of a 4/8ns echo as a function of the digital scale of our AWG. (Bis-trityl **1**). Note that starting from around 0.3, the saturation of the TWT becomes significant.

2. **Set up the optimal phase of the spin-lock** with a sequence $\pi/2 - Lock - \tau - \pi - \tau$- echo. In principle, this should not be necessary when using an AWG. In fact, we checked that the phase cycling in our setup works nearly perfectly when simply programming the phases. Additionally, in a two-pulse echo, the phase of the signal perfectly follows the expected phase shift of the coherence order pathway. If this is the case, one would expect that one could simply apply the spin-lock pulse at 90 degrees to the $\pi/2$ pulse, but experimentally, we observe that this is not the case, see Fig. S2. We suspect that phase transients of the pulses due to the resonator are responsible for this behavior.

3. **Measure $T_{1\rho}$** with the sequence $\pi/2 - Lock - \tau - \pi - \tau$ -echo (SL + echo) with variable length of the spin-lock pulse. Note that a spin-locked echo ($\pi/2 - \tau - Lock - \tau$ - echo, SLE) gives very different results in the case of trityl radicals at low temperatures, see the following Fig. S3. Note also that in the case of the SLE, the decay depends on the interpulse delay. Curiously, one can again see that the maximal echo intensity is not achieved when the spin-lock pulse corresponds to a π pulse, but when it corresponds to a $\pi/2$ or $3\pi/2$ pulse. The red traces in Fig.

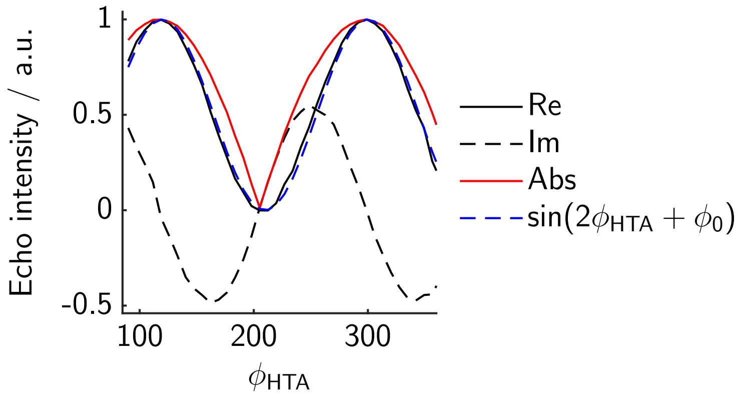

Fig. S2: Echo intensity after a sequence $\pi/2 - Lock - \tau - \pi - \tau$- echo as a function of the digital phase of the spin-lock pulse relative to the first pulse. The length of the spin-lock pulse was $5\mu s$. The highest expected absolute intensity of the echo is at around 120 degrees, while one would expect it around 90 degrees.

S3 correspond to a nutation of the second pulse. Obviously, not only the fundamental oscillation with the Rabi frequency of slightly more than 100 MHz is present, but also an oscillation which is twice as fast. This is due to the fact that a significant part of the echo reduction of the two-pulse echo is due to the dipolar coupling, which is refocused by a solid echo, not a Hahn echo. The solid echo requires a $\pi/2$ pulse. Note however, that the solid echo does not fully refocus the dipolar coupling in the presence of significant offsets compared to the interpulse delay.

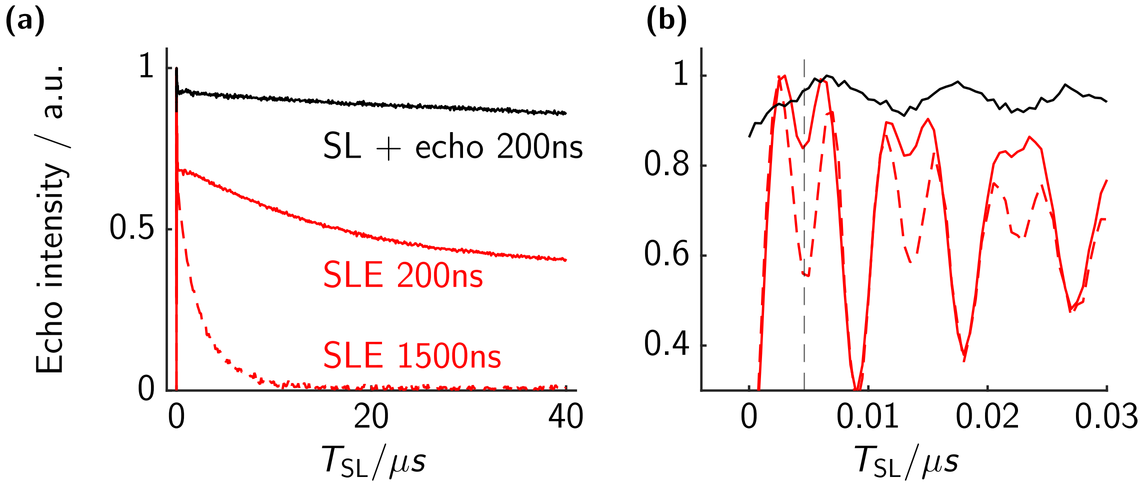

Fig. S3: Measurement of $T_{1\rho}$ with different sequences and different settings. See main text for more details. **(b)** is a zoom of **(a)**.

4. **Record a frequency-stepped dressed spin resonance spectrum.** In order to set the frequency of the phase-modulation, we need to know the Rabi frequency of the spin-lock pulse. It is also useful to know the distribution of the Rabi frequency. One possibility to obtain this information is to use the basic sequence introduced in the main text, but with only a single period of phase-modulation. The amplitude of the phase-modulation should be rather small (*e.g.* 0.02 radians), and the pulse should be rather long (*e.g.* 1 μs.) in order to avoid significant broadening. The

frequency of the phase-modulation is then stepped, and the echo intensity recorded. This is shown in black in Fig. S4. As a comparison, we also show the Fourier transform of a simple nutation experiment in red. The frequency of the phase-modulation for all successive steps was then set to the maximum of the dressed spin resonance spectrum.

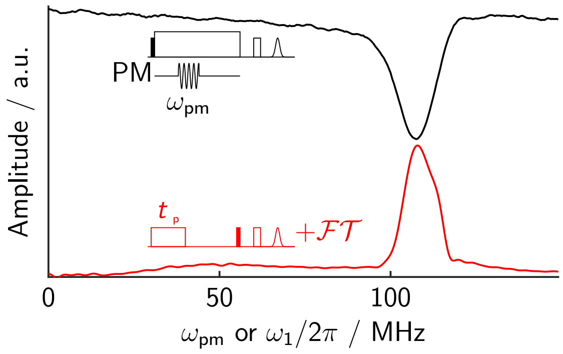

Fig. S4: Frequency-stepped dressed-spin resonance spectrum (black, $a_{pm} = 0.02$, phase pulse length 1 μs). And Fourier transform of a nutation experiment at full power (red). Insets show the pulse sequences. PM denotes the phase modulation during the spin-lock, not an additional channel. Based on this spectrum, a PM frequency of $\omega_{PM}/(2\pi)$ =108 MHz was chosen.

5. **Set up the phase-modulation amplitude and phase-pulse length** Now that the frequency of the phase modulation (ω_{PM}) is set, we can setup the phase-pulse amplitude and length. We can do this by using the same sequence as in the main text, but with only one phase-pulse with fixed modulation frequency. By changing the length of the phase-modulation pulse one obtains a nutation curve in the nutating frame. Fig. S5 shows such nutation curves for $a_{PM} = 0.2$ (grey and black) and $a_{PM} = 0.3$ (red and light red). Interestingly, one can see an overlaid oscillation, which depends on the phase of the phase modulation (ϕ_2). This additional oscillation is due to the breakdown of the rotating wave approximation in the nutating frame and becomes more pronounced with larger phase-modulation intensity. In this case we used the minimum of the phase-pulse nutation curve as a dressed π-pulse length (around 43 ns for $a_{PM} = 0.3$).

6. **Set up the timings for the dressed echo and check the phase-phase cycling.** The sequence from the main text is repeated here in Fig. S6 (a). The dressed echo cannot be acquired continuously. Therefore, we need to check whether the timings of the sequence are correct. In order to do this, we use a fixed spin-lock length T_{SL}, a fixed τ_0 and τ_1 and changed τ_2 step-by-step. A trace with a two-step phase-cycle of $\phi_1 = [(+)0, (-)180]$ and $\phi_2 = \phi_3 = \phi_4 = 0$ is shown in red in Fig. S6 (b). The highest intensity indicates the dressed echo where $\tau_2 = \tau_1$ (plus the length of the first phase pulse, t_p). In addition, one can clearly see additional, *crossing* echoes, and a “dressed FID” around $\tau_2 = 0$. We realized that the position of these crossing echoes depend on the choice of τ_0. This indicates that there is some dressed coherence present during τ_0. This is not very surprising, because the first $\pi/2$ pulse will never be perfect, and some magnetization will be orthogonal to the effective field of the spin-lock. In order to get rid of these crossing echoes, one can cycle the phase of the phase-pulses ϕ_{2-4} in analogy to a bare spin echo, with the complication that at the beginning of the first dressed pulse (after τ_0),

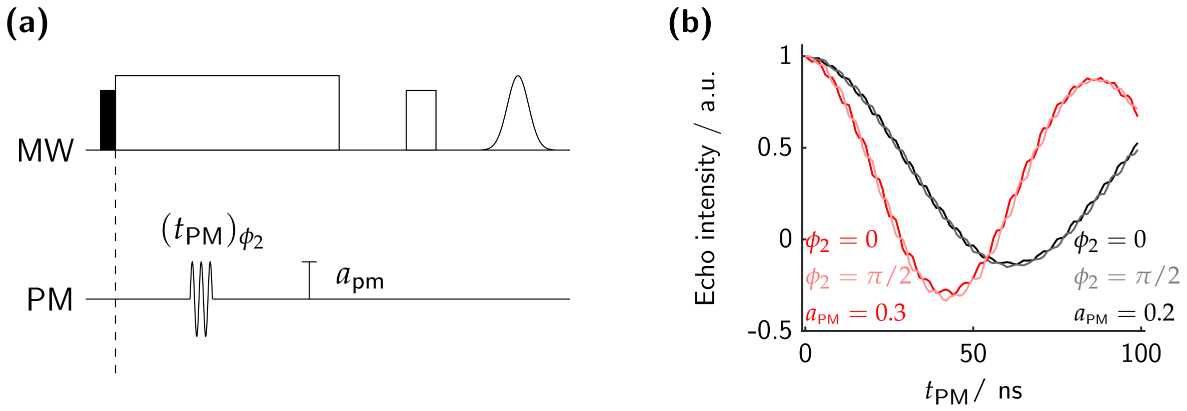

Fig. S5: **(a)** Pulse sequence to set up the dressed pulses. "PM" denotes the phase-modulation during the spin-lock, *not* and additional channel. **(b)** Phase-pulse nutation curves obtained with fixed ω_{PM} but different intensities and phases. The minima of the curves indicate the length of the dressed π pulses for a given intensity a_{PM}.

there is already dressed coherence present. The black trace in Fig. S6 (b) shows the result of the measurement with the 16-step nested phase cycle $\phi_1 = [(+)0, (-)180]$, $\phi_2 = [(+)0, (-)180]$, $\phi_3 = [(+)0, (-)90, (+)180, (-)270]$, which essentially gets rid of all crossing echoes observed in our case. Note that this cycle relies on the fact that dressed coherence decays relatively quickly after the last phase-pulse due to inhomogeneities, and that it does not get refocused after that point. The exact implementation of the phase-modulation and thus the meaning of the phases are given in the next section.

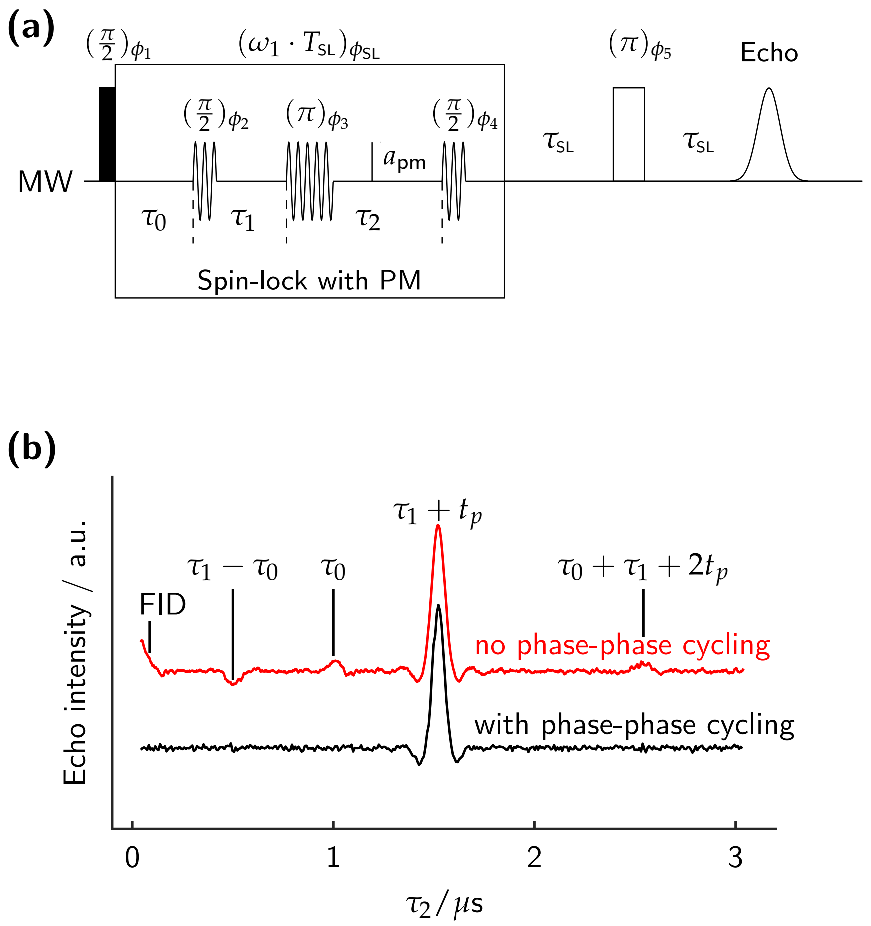

Fig. S6: **(a)** Pulse sequence of the dressed spin echo. **(b)** Dressed echo with fixed τ_0=1μs and τ_1=1.5μs and variable τ_2. Red: $\phi_1 = [(+)0, (-)180]$. Clear crossing echoes are visible. Black: Nested cycle $\phi_1 = [(+)0, (-)180]$, $\phi_2 = [(+)0, (-)180]$, $\phi_3 = [(+)0, (-)90, (+)180, (-)270]$. No crossing echoes visible anymore.

S.3 Implementation details

On our homebuilt AWG based spectrometer, the actual pulse waveform that reaches the sample is generated by IQ-mixing the AWG waveform (IF) with a local oscillator (LO). As an example, we use a Q-band phase-modulated pulse. The LO in this case is around 33.3 GHz. The center frequency of the pulse generated by the AWG is always around 1.5 GHz in our setup. This leads to a final pulse frequency of 33.3+1.5 = 34.8 GHz that reaches the resonator and the spins, as seen in Fig. 3(a), 4(a) and 5(a) in the main text. Apart from the constant value of around 1.5 GHz, we do not use any frequency modulation (FM) in this work ($FM(t) = 1.5$ GHz). The amplitude is also constant during the spin-lock. So let us look now at the phase modulation (PM), which is illustrated in Fig. S7 in red. At most times during the spin-lock, there is no phase modulation. But during the “phase pulses”, we turn on the sinusoidal PM. It is best to think about these phase pulses in relation to a hypothetical “carrier”. The frequency of this carrier is the dressed spin resonance frequency $\omega_{\mathrm{PM}} \approx \omega_1$ that we determined before in Fig. S4. The phase of this carrier is arbitrarily set to 0 at the beginning of the spin-lock pulse. In our experiment, all three phase pulses have the same frequency as the carrier, but each phase pulse might be phase shifted. This is seen in the example below. The first pulse has a phase $\phi_2 = 0$, which means that the phase is the same for the hypothetical carrier and the phase pulse. The second phase pulse is phase shifted by $\phi_3 = \pi$, and the third one by $\phi_4 = \pi/2$. By cycling the phases of the phase pulses, we can select certain dressed coherence order pathways, in analogy to the bare spin case. Of course the amplitude of the phase modulation (0.3 rad in the example shown here) as

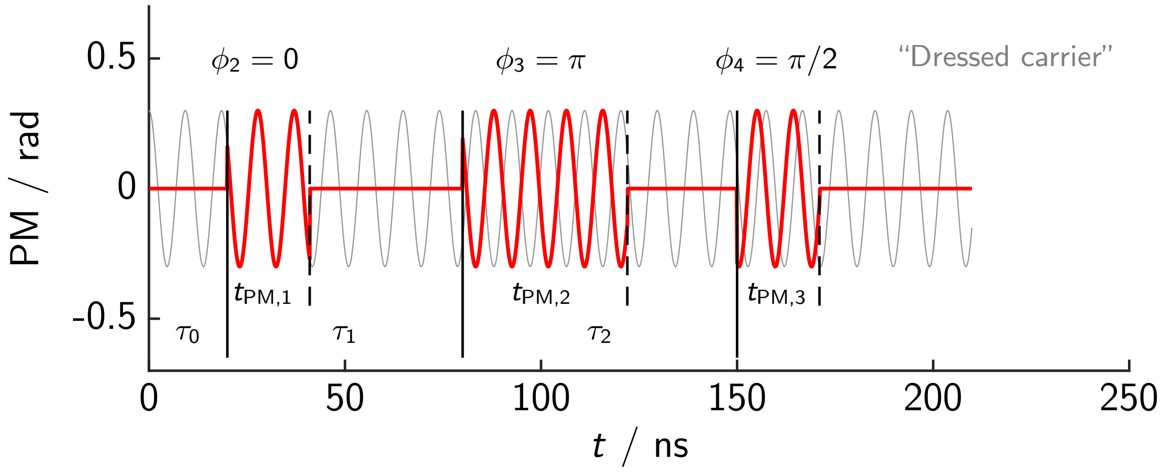

Fig. S7: Illustration of the

well as the length of each phase pulse can be chosen at will. Mathematically, the PM is given by

$$PM(t) = \begin{cases} a_{\mathrm{PM},1} \cos\left(\omega_{\mathrm{PM}} t + \phi_2\right) & \text{for } \tau_0 < t < \tau_0 + t_{\mathrm{PM},1} \\ a_{\mathrm{PM},2} \cos\left(\omega_{\mathrm{PM}} t + \phi_3\right) & \text{for } (\tau_0 + \tau_1) < t < (\tau_0 + \tau_1 + t_{\mathrm{PM},2}) \\ a_{\mathrm{PM},3} \cos\left(\omega_{\mathrm{PM}} t + \phi_4\right) & \text{for } (\tau_0 + \tau_1 + \tau_2) < t < (\tau_0 + \tau_1 + \tau_2 + t_{\mathrm{PM},3}) \\ 0 & \text{otherwise} \end{cases} \tag{45}$$

The total final spin-lock waveform generated by the two channels of the AWG (real and imaginary) is then given by

$$
\begin{aligned}
f(t) =& AM(t) \cdot \left[\cos\left(2\pi \cdot \int_0^t FM(\tau)\mathrm{d}\tau + PM(t) + \phi_{\mathrm{SL}} \right) \right. \\
& \left. + i\sin\left(2\pi \cdot \int_0^t FM(\tau)\mathrm{d}\tau + PM(t) + \phi_{\mathrm{SL}} \right] \right) \quad , \qquad (46)
\end{aligned}
$$

where ϕ_{SL} is the overall phase of the spin-lock pulse. We omitted the possible corrections for phase and amplitude for the real and imaginary part.

S.4 Additional experimental data

S.4.1 Dipolar echo envelope modulation: Influence of pulse length

As mentioned in the main text, the dipolar coupling is already visible in the normal Hahn echo decay. Fig. S8 shows these echo decays for the two different bis-trityl compounds, and with different pulse lengths. For both bis-trityls, we see and additional oscillation when very short pulses (4/8 ns) are used. With 100/200 ns pulses, the modulation intensity is reduced. In the case of bis-trityl **2** (5.3 nm) and 4/8 ns pulses, the echo modulation even crosses zero. The presence of these oscillations complicates the analysis of the relaxation data.

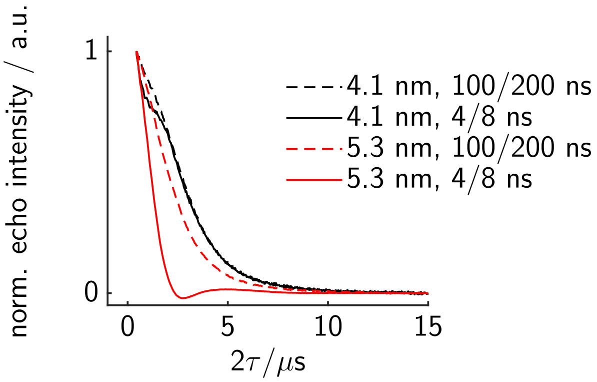

Fig. S8: Hahn echo decay for bis-trityl **1** (4.1 nm) and bis-trityl **2** (5.3 nm) acquired with different pulse lengths. The oscillations are due to the dipolar coupling.

S.4.2 Solvent deuteration: dOTP *vs* OTP

We measured the Hahn echo decay of bis-trityl **1** in *ortho*-terphenyl (OTP) and its deuterated variant (dOTP). Surprisingly, we observed no difference between the two, see Fig. S9. Most likely, the protons in the trityl itself are responsible for the relaxation. Unfortunately, we do not (yet) have the fully deuterated variants of the used trityl moieties in order to test this hypothesis.

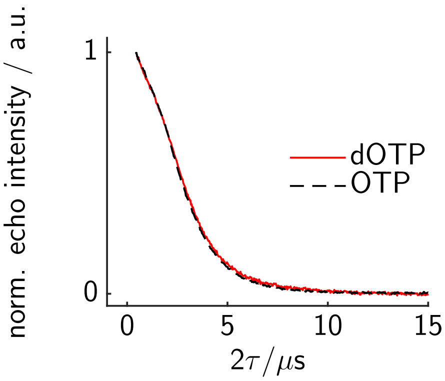

Fig. S9: Hahn echo decay of bis-trityl **1** acuqired with 100/200 ns pulses with OTP and dOTP as solvents. No significant difference can be observed between the two.

S.4.3 SIFTER data for bis-trityls 1 and 2

The SIFTER traces for bis-trityls **1** and **2** and corresponding distance distributions including a sensitvity analysis regarding the background and additional noise are shown in Fig. S10. The fitted background was included in the kernel function[1]. The distance distributions show the expected distances of around 4.1 and 5.3 nm. The oscillations in the case of bis-trityl **1** are quite damped compared to other model systems, and also compared to the dressed echo traces shown in the main text. It is possible that the compound shows strong or intermediate coupling effects for some orientations.

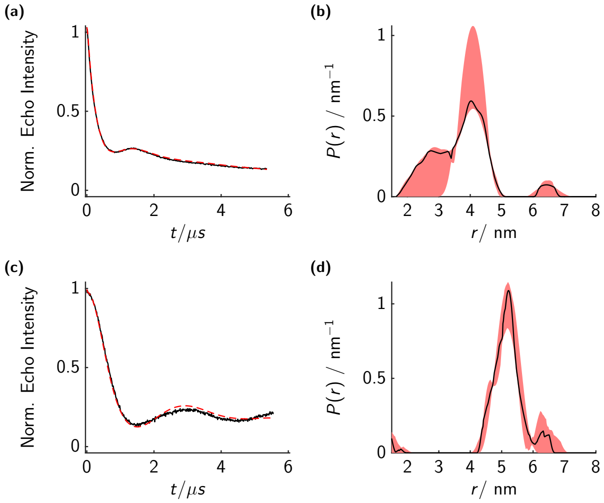

Fig. S10: SIFTER data for bis-trityls **1** and **2**. (**a**) Experimental SIFTER trace (black), symmetrized around the zero-time. Best fit including background in red. (**b**) Corresponding distance distributions obtained with the DeerLab API (`https://github.com/luisfabib/deerlab`). Black curve shows the median values for the probability densities. Red areas span from the 0.25 to 0.75 quantile.

S.5 Additional data for figures in the main article

Here we give detailed information on each figure. This includes information about raw data and processing scripts which are deposited online with a permanent DOI: `10.5281/zenodo.3703053`. Since our home-built spectrometer is run *via* MATLAB© (The MathWorks, Inc), even the raw data are stored in `.mat` files. Some scripts use functions from *EasySpin*[4]. Since *EasySpin*, written in MATLAB, is the most widely used simulation library in the EPR community, we believe that a majority of readers will be able to read and run the scripts we provide. Note that the `.mat` files can also be read into the free software R, with the help of the library 'R.matlab' (https://cran.r-project.org/web/packages/R.matlab/).

Fig. 3 (a)

Q-Band chirp echo Fourier transform EPR spectrum of mono-trityl **7**. 200 μM in dOTP. 6 μl of the solution in a 1.6 mm outer diameter tube. Temperature: 50 K. Pulse sequence: 200/100ns two-pulse chirp echo. Linear chirp pulses with total sweep width of 300 MHz, centered at the center of the resonator (34.8 GHz) and 20 ns rise time were used. The FM function was adjusted to compensate the resonator profile. Interpulse delay 2 μs (start-to-start of pulses). A symmetric 3.6 μs Chebyshev window with 100 dB relative sidelobe attenuation was applied to the digitally down-converted echo. A Fourier transform then yielded the spectrum. Magnetic field: 1241.4 mT (calibrated with DPPH). Shot repetition time 10 ms. 100 shots. 10 averages. $[(+)0, (-)180]$ phase cycle for the first pulse.
Raw data and processing scripts: Matlab processing and simulation: `spectrum_monomer.m`
raw file: `20191106_1239_chirp_echo_dummy.mat`

Fig. 3 (b)

Relaxation measurements for mono-trityl **7**. 200 μM in dOTP. 6 μl of the solution in a 1.6 mm outer diameter tube. Temperature: 50 K. Magnetic field: 1241.4 mT.
T_m measurement: Two-pulse echo ($\pi/2 - \tau - \pi - \tau -$ echo) with 100/200 ns pulses. $[(+)0, (-)180]$ phase cycle for the first pulse. Inter-pulse delay varied from 200 ns to 16552 ns in steps of 32 ns (512 points in total). Note that the trace was to long and was thus cut for processing and plotting. Shot repetition time 30 ms. 10 shots per point. 1 average.
$T_{1\rho}$ measurement: $\pi/2 -$ lock $- \tau - \pi - \tau -$echo. $(\pi/2)/\pi$ =4/8 ns. τ=200 ns. Spin-lock Rabi frequency around 100 MHz. $[(+)0, (-)180]$ phase cycle for the $\pi/2$ pulse, $[(+)0, (+)180]$ phase cycle for the π pulse. Fixed phase for the spin-lock, but always orthogonal to the first pulse. Spin-lock length varied from 0 ns to 100 ns in steps of 0.5 ns, and from 200 ns to 40 μs in steps of 100 ns (600 points in total). Shot repetition time 30 ms. 5 shots per point. 1 average.
$T_{2\rho}$ measurement: Dressed spin echo, see previous sections for detailed definitions. Dressed $(\pi/2)/\pi$ pulse lengths 21/42 ns, with an amplitude of 0.3 rad. Total spin-lock length 35 μs. Modulation frequency of 99 MHz. τ_0 =2 μs, τ_1 varied from 22 ns to 14390 ns in steps of 32 ns (450 points in total). Phase cycle and phase-phase cycle as introduced above. Shot repetition time 30 ms. 1 shot per point. 300 averages (overkill, was simply overnight). All other parameters as for the $T_{1\rho}$ measurement.
Raw data and processing scripts:

Matlab processing and simulation: `relaxation_monomers.m`
raw file T_m measurement: `20200203_1538_2pecho_ESEEM.mat`
raw file $T_{1\rho}$ measurement: `20200203_1549_spin_locked_echo_t1rho.mat`
raw file $T_{2\rho}$ measurement: `20200203_1700_spin_locked_phase_echo_mod`

Fig. 4 (a)

Q-Band chirp echo Fourier transform EPR spectrum of bis-trityl **1**. 100 μM in dOTP. 6 μl of the solution in a 1.6 mm outer diameter tube. Temperature: 50 K. Pulse sequence: 200/100ns two-pulse chirp echo. Linear chirp pulses with total sweep width of 300 MHz, centered at the center of the resonator (34.8 GHz) and 20 ns rise time were used. The FM function was adjusted to compensate the resonator profile. Interpulse delay 950 ns (start-to-start of pulses). A symmetric 3.6 μs Chebyshev window with 100 dB relative sidelobe attenuation was applied to the digitally down-converted echo. A Fourier transform then yielded the spectrum. Magnetic field: 1241.4 mT (calibrated with DPPH). Shot repetition time 10 ms. 100 shots. 1 average. $[(+)0, (-)180]$ phase cycle for the first pulse.
Raw data and processing scripts: Matlab processing and simulation: `spectrum_4p1nm.m`
raw file: `20190729_1134_chirp_echo_dummy.mat`

Fig. 4 (b)

Relaxation measurements for bis-trityl **1**. 100 μM in dOTP. 6 μl of the solution in a 1.6 mm outer diameter tube. Temperature: 50 K. Magnetic field: 1241.4 mT.
T_m measurement: Two-pulse echo ($\pi/2 - \tau - \pi - \tau -$ echo) with 100/200 ns pulses. $[(+)0, (-)180]$ phase cycle for the first pulse. Inter-pulse delay varied from 200 ns to 8376 ns in steps of 16 ns (512 points in total). Shot repetition time 30 ms. 10 shots per point. 1 average.
$T_{1\rho}$ measurement: $\pi/2 -$ lock $- \tau - \pi - \tau -$echo. $(\pi/2)/\pi$ =4/8 ns. τ=200 ns. Spin-lock Rabi frequency around 100 MHz. $[(+)0, (-)180]$ phase cycle for the $\pi/2$ pulse, $[(+)0, (+)180]$ phase cycle for the π pulse. Fixed phase for the spin-lock, but always orthogonal to the first pulse. Spin-lock length varied from 0 ns to 100 ns in steps of 0.5 ns, and from 200 ns to 40 μs in steps of 100 ns (600 points in total). Shot repetition time 30 ms. 5 shots per point. 1 average.
Raw data and processing scripts:
Matlab processing and simulation: `relaxation_4p1nm.m`
raw file T_m measurement: `20191101_1415_2pecho_ESEEM.mat`
raw file $T_{1\rho}$ measurement: `20190819_1706_spin_locked_echo_t1rho.mat`

Fig. 4 (c) and (d)

Dressed echo decay/modulation for bis-trityl **1**. See previous sections for detailed definitions. 100 μM in dOTP. 6 μl of the solution in a 1.6 mm outer diameter tube. Temperature: 50 K. Magnetic field: 1241.4 mT. Dressed $(\pi/2)/\pi$ pulse lengths 21/42 ns, with an amplitude of 0.34 rad. Total spin-lock length 25 μs. Modulation frequency of 108 MHz. τ_0 =1 μs, τ_1 varied from 22 ns to 8997 ns in steps of 25 ns (360 points in total). Phase cycle and phase-phase cycle as introduced above. Shot repetition

time 30 ms. 5 shot per point. 63 average. All other parameters as for the $T_{1\rho}$ measurement. Background correction with a stretched exponential and a Fourier transform gives the spectrum.
Raw data and processing scripts:
Matlab processing and simulation: `dressed_echo_modulation_4p1nm.m`
raw file: `20190820_1736_spin_locked_phase_echo_mod.mat`

Fig. 5 (a)

Q-Band chirp echo Fourier transform EPR spectrum of bis-trityl **2**. 100 μM in dOTP. 6 μl of the solution in a 1.6 mm outer diameter tube. Temperature: 50 K. Pulse sequence: 200/100ns two-pulse chirp echo. Linear chirp pulses with total sweep width of 300 MHz, centered at the center of the resonator (34.8 GHz) and 20 ns rise time were used. The FM function was adjusted to compensate the resonator profile. Interpulse delay 950 ns (start-to-start of pulses). A symmetric 3.6 μs Chebyshev window with 100 dB relative sidelobe attenuation was applied to the digitally down-converted echo. A Fourier transform then yielded the spectrum. Magnetic field: 1241.4 mT (calibrated with DPPH). Shot repetition time 10 ms. 100 shots. 1 average. $[(+)0,(-)180]$ phase cycle for the first pulse.
Raw data and processing scripts: Matlab processing and simulation: `spectrum_5p3.m`
raw file: `20191101_1155_chirp_echo_dummy.mat`

Fig. 5 (b)

Relaxation measurements for bis-trityl **2**. 100 μM in dOTP. 6 μl of the solution in a 1.6 mm outer diameter tube. Temperature: 50 K. Magnetic field: 1241.4 mT.
T_m measurement: Two-pulse echo ($\pi/2 - \tau - \pi - \tau -$ echo) with 100/200 ns pulses. $[(+)0,(-)180]$ phase cycle for the first pulse. Inter-pulse delay varied from 200 ns to 8376 ns in steps of 16 ns (512 points in total). Shot repetition time 30 ms. 10 shots per point. 1 average.
$T_{1\rho}$ measurement: $\pi/2 - \text{lock} - \tau - \pi - \tau -$echo. $(\pi/2)/\pi$ =4/8 ns. τ=200 ns. Spin-lock Rabi frequency around 100 MHz. $[(+)0,(-)180]$ phase cycle for the $\pi/2$ pulse, $[(+)0,(+)180]$ phase cycle for the π pulse. Fixed phase for the spin-lock, but always orthogonal to the first pulse. Spin-lock length varied from 0 ns to 100 ns in steps of 0.5 ns, and from 200 ns to 40 μs in steps of 100 ns (600 points in total). Shot repetition time 30 ms. 5 shots per point. 1 average.
Raw data and processing scripts:
Matlab processing and simulation: `relaxation_5p3nm.m`
raw file T_m measurement: `20191101_1336_2pecho_ESEEM.mat`
raw file $T_{1\rho}$ measurement: `20191101_1058_spin_locked_echo_t1rho.mat`

Fig. 5 (c) and (d)

Dressed echo decay/modulation for bis-trityl **2**. See previous sections for detailed definitions. 100 μM in dOTP. 6 μl of the solution in a 1.6 mm outer diameter tube. Temperature: 50 K. Magnetic field: 1241.4 mT. Dressed $(\pi/2)/\pi$ pulse lengths 22/44 ns, with an amplitude of 0.3 rad. Total spin-lock length 35 μs. Modulation frequency of 108 MHz. τ_0 =1 μs, τ_1 varied from 22 ns to 14390 ns in steps

of 32 ns (450 points in total). Phase cycle and phase-phase cycle as introduced above. Shot repetition time 30 ms. 5 shot per point. 56 average. All other parameters as for the $T_{1\rho}$ measurement.
Background correction with a stretched exponential and a Fourier transform gives the spectrum.
Raw data and processing scripts:
Matlab processing and simulation: `dressed_echo_modulation_5p3nm.m`
raw file: `20190821_1636_spin_locked_phase_echo_mod.mat`

Fig. 6 (a) to (d): Simulations
The simulation scripts are named *runsim_*.m*. Where the asterisk describes the distance, offset distributions and Rabi frequencies. The actual simulation is done by the function *spinlock.m* . The corresponding plotting scripts are named *plot_*.m*. (But you have to run the simulations first such that the results are saved).

References

1 Fábregas Ibáñez, L. and Jeschke, G.: Optimal background treatment in dipolar spectroscopy, Phys. Chem. Chem. Phys., 22, 1855–1868, https://doi.org/10.1039/C9CP06111H, 2020.

2 Henstra, A., Dirksen, P., Schmidt, J., and Wenckebach, W. T.: Nuclear spin orientation via electron spin locking (NOVEL), Journal of Magnetic Resonance (1969), 77, 389–393, https://doi.org/10.1016/0022-2364(88)90190-4, 1988.

3 Meyer, A., Jassoy, J. J., Spicher, S., Berndhäuser, A., and Schiemann, O.: Performance of PELDOR, RIDME, SIFTER, and DQC in measuring distances in trityl based bi- and triradicals: Exchange coupling, pseudosecular coupling and multi-spin effects, Physical Chemistry Chemical Physics, 20, 13 858–13 869, https://doi.org/10.1039/c8cp01276h, 2018.

4 Stoll, S. and Schweiger, A.: EasySpin, a comprehensive software package for spectral simulation and analysis in EPR, Journal of Magnetic Resonance, 178, 42–55, https://doi.org/10.1016/j.jmr.2005.08.013, 2006.
